# Lighting Up Biology: GFP-Inspired Fluorescent Probes for Sensing and Imaging

**DOI:** 10.1021/jacs.5c16404

**Published:** 2026-08-11

**Authors:** Qianfang Qiu, Luling Wu, Robin R. Groleau, Xuanyu Zhu, Tony D. James, Chusen Huang

**Affiliations:** † The Education Ministry Key Laboratory of Resource Chemistry, Shanghai Key Laboratory of Rare Earth Functional Materials, Shanghai Frontiers Science Center of Biomimetic Catalysis, and Shanghai Municipal Education Committee Key Laboratory of Molecular Imaging Probes and Sensors, Department of Chemistry, 12544 Shanghai Normal University, 100 Guilin Road, Shanghai 200234, China; ‡ State Key Laboratory of Analytical Chemistry for Life Science, School of Chemistry, 12581 Nanjing University, 163 Xianlin Avenue, Nanjing 210023, China; § Department of Chemistry, 1555 University of Bath, Bath BA2 7AY, U.K.; ∥ Department of Life Sciences, University of Bath, Bath BA2 7AY, U.K.; ⊥ School of Chemistry and Chemical Engineering, Henan Normal University, Xinxiang 453007, China

## Abstract

Green fluorescent protein (GFP) chromophore analogues are derived from the core structure of the GFP chromophore, *p*-hydroxybenzylideneimidazolidinone (HBDI). Appealing and readily tunable, HBDI analogues have been adapted for a range of sensing and imaging applications, as is the focus of this perspective. First, common methods for their synthesis will be presented, followed by a discussion of the unique structural and photophysical properties of HBDI derivatives. Structural modifications resulting in tunable photophysical properties will then be highlighted. Finally, this perspective will present representative examples of the use of HBDI analogues for sensing and imaging in biological sciences. This perspective highlights key design strategies, modes of activation, and the associated advantages, limitations, and opportunities of HBDI analogues, illustrated by representative applications in studying biomolecules, cellular processes, and disease biomarkers.

## Graphical abstract

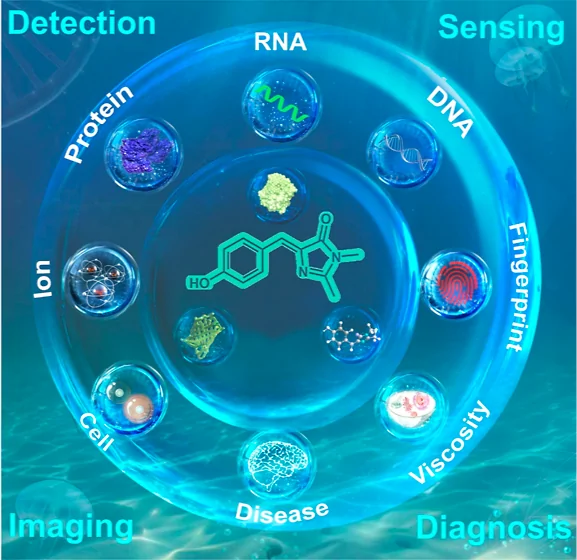

## 1 Introduction

Following its original discovery in 1962 ^1^ and subsequent expression and characterization in 1992,^2^ green fluorescent protein (GFP) has become a widely used tool for the fluorescence-based visualization of biological processes. Owing to its favorable photochemical properties, GFP enables in situ tracking of the localization, dynamics, and interactions of biomolecules in living cells and organisms.^3^ Now an indispensable tool in the biosciences, GFP and its variants have enabled numerous discoveries, as recognized by the 2008 Nobel Prize in Chemistry “for the discovery and development of the green fluorescent protein”.^1^ Continued engineering of GFP variants has further expanded their utility, with examples such as reversibly photoswitchable fluorescent proteins that have advanced super-resolution microscopy.^4,5^ These developments have also inspired the design of small-molecule fluorophores derived from GFP’s core chromophore, which seek to retain environmental sensitivity while offering enhanced synthetic tunability and compatibility with live-cell applications.

The fluorescence emission of GFP originates from its *p*-hydroxybenzylidenemidazolidinone chromophore (**HBDI**, Figure 1) which forms autocatalytically from the tripeptide motif Ser-Tyr-Gly at positions 65–67.^6^ This chromophore framework is retained across GFP variants,^7^ enabling fluorescence emission from cyan to red via relatively minor structural and conformational modifications.^8–10^ As a protein-embedded chromophore, **HBDI** formation requires precise folding, cyclization, and oxidative post-translational processing following gene expression,^6,11^ resulting in a substantial temporal burden in vivo, typically ca. 2–4 h.^12^ In addition, variability in protein expression levels can introduce challenges in quantitative reliability.^13^ Although extensive efforts have been made to mitigate these limitations, for example through enhanced expression and the development of brighter, faster maturing variants such as EGFP (enhanced GFP),^14^ protein-based fluorophores remain intrinsically constrained by their biosynthetic and maturation requirements.^15,16^

**Figure 1:**
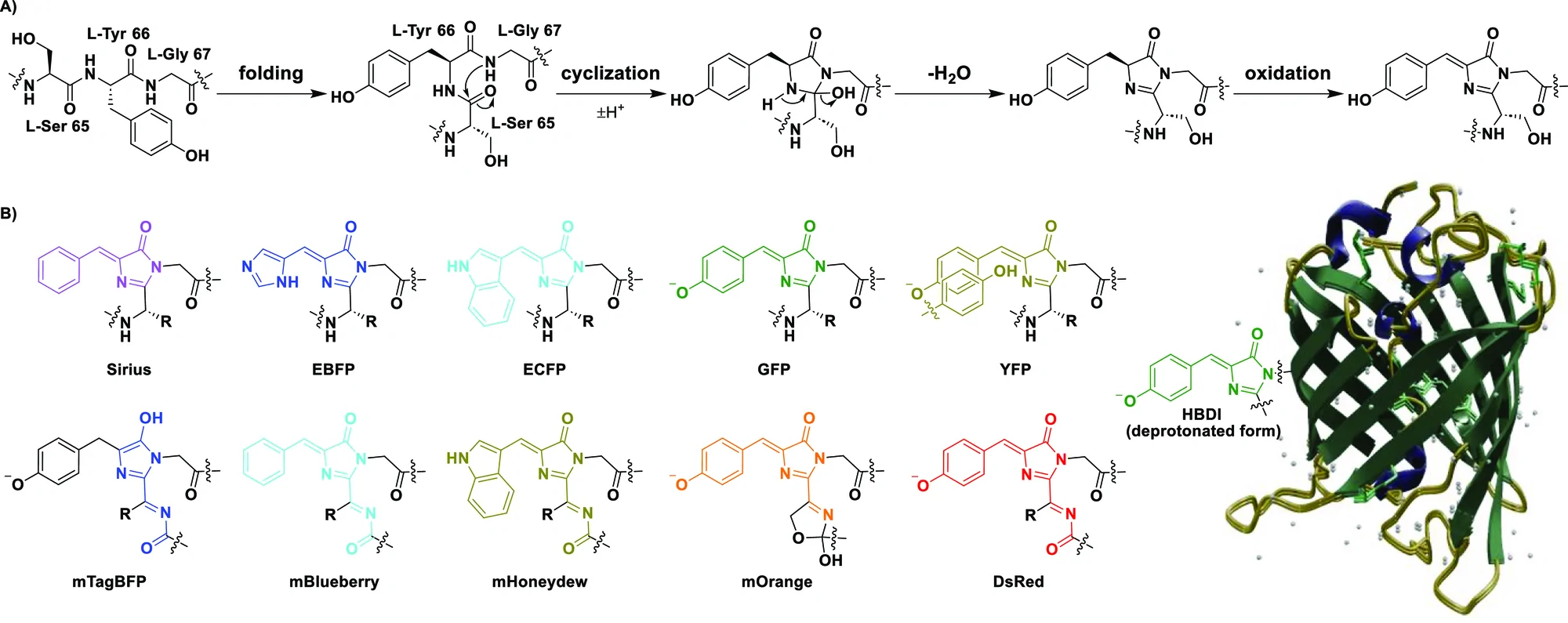
(A) Biosynthetic formation of the green fluorescent protein (GFP) chromophore from the tripeptide Ser^65^–Tyr^66^–Gly.^67^ (B) Core chromophore structures of representative fluorescent proteins. The R group denotes variable side chains. All chromophores are depicted in their deprotonated (anionic) form, representing the emissive states relevant for fluorescence under physiological conditions.^22^ The β-barrel schematic on the right is based on the X-ray crystal structure of wild-type GFP from the Protein data bank (ID:1EMA^23^).

Small-molecule organic fluorophores typically avoid these biosynthetic and maturation constraints offering easier production and chemical modification, along with improved stability and reliability for biological applications. A wide array of synthetic fluorescent dyes is available, including fluoresceins,^17^ rhodamines,^18^ cyanine dyes,^19^ coumarins,^20^ and BODIPYs.^21^ Nevertheless, there remains strong demand for new and more versatile fluorescence-based systems. Many conventional dyes suffer from limitations such as small Stokes shifts, limited photostability, low environmental sensitivity, or high background fluorescence, which can hinder their utility for advanced microscopic applications. Although the native GFP chromophore is essentially nonfluorescent in isolation, strategic structural modifications have enabled the development of biomimetic small-molecule fluorophores that preserve key photophysical features while gaining cell permeability, synthetic tunability, and compatibility with live-cell labeling.

When confined within the interior of GFP’s β-barrel, the **HBDI** chromophore displays strong fluorescence, with its fluorescence quantum yield (Φ) reaching up to 80%.^9^ In contrast, only very weak fluorescence is observed outside of the protein environment,^24,25^ although the emission can be partially restored upon freezing.^26^ This pronounced enhancement arises from two synergistic effects imposed by the β-barrel scaffold: (i) conformational rigidification, which suppresses nonradiative decay pathways, including torsional motion and E/Z isomerization of the exocyclic C=C bond, as well as rotation about the phenol–imidazolinone linkage.^27–30^ (ii) Electrostatic stabilization of the deprotonated (anionic) chromophore, with the anionic state being the dominant species under physiological conditions (as depicted in Figure 1B). These tunable photophysical features have enabled the development of a broad range of GFP mimetic fluorescent probes, which will be discussed in detail herein.^31–33^

Typical applications of GFP-chromophore-inspired fluorescent probes include RNA sensing,^34^ protein aggregation detection,^35^ G-quadruplex recognition,^36^ and probing of cellular viscosity.^37^ Owing to the relatively facile synthesis of the **HBDI** motif (vide infra), production and functionalization of this scaffold through synthetic chemistry is straightforward, enabling rational design of new fluorophores with tailored photophysical properties. Crucially, synthetic modification provides extensive freedom to fine-tune the emission wavelength, brightness, and stability.

Relative to widely used chromophores such as fluoresceins, rhodamines, cyanine dyes, coumarins, and BODIPYs, GFP and **HBDI**-inspired chromophore analogues are at an early stage of development. As a result, systematic exploration of their performance optimization and application potential is increasingly important. Understanding the photophysical mechanisms of the GFP chromophore, particularly its environmental sensitivity and rigidification-induced emission, therefore provides a valuable blueprint for the design of synthetic fluorescent probes. This perspective will explore recent advances in sensing and imaging applications of GFP-inspired fluorophores and analyzes how their design is rooted in the biomimicry of native chromophore function. It will focus on translating core principles, such as rigidification to suppress nonradiative decay, modulation of the electronic structure, and tuning chromophore protonation/deprotonation states, into chemical modifications that enable context-dependent fluorescence responses. This bioinspired approach provides a useful basis for developing optical tools tailored to biological and chemical sensing.

## 2 Synthetic Strategies for Constructing the Core Structures of GFP Chromophores and Analogues

Various synthetic strategies can be employed to synthesize GFP-type chromophores (Scheme 1). Erlenmeyer–Plöchl reactions^38,39^ can be used, combining hippuric or aceturic acid derivatives with aldehydes in the presence of acetic anhydride and sodium acetate (Scheme 1A). This approach requires only two steps, proceeding via an azlactone or oxazolone **1-1**, reaction of which with an amine provides the final imidazolinone chromophore with different R_1_ and R_2_. Though straightforward, the Erlenmeyer–Plöchl approach suffers from low yields in the second step. On reaction of **1-1** with the amine, ring-opening to bis-amide intermediate **1-3** occurs which requires ring-closure to product **1-2**. This ring-closing process is very sensitive to steric hindrance,^40^ limiting both yield and functional tolerance, excluding for instance the use of pyrrole-bearing building blocks.^41^ Additionally, formamides are not tolerated, so the reaction does not proceed when R_2_ = H, instead undergoing condensation to the corresponding isonitrile.^40^

**Scheme 1:**
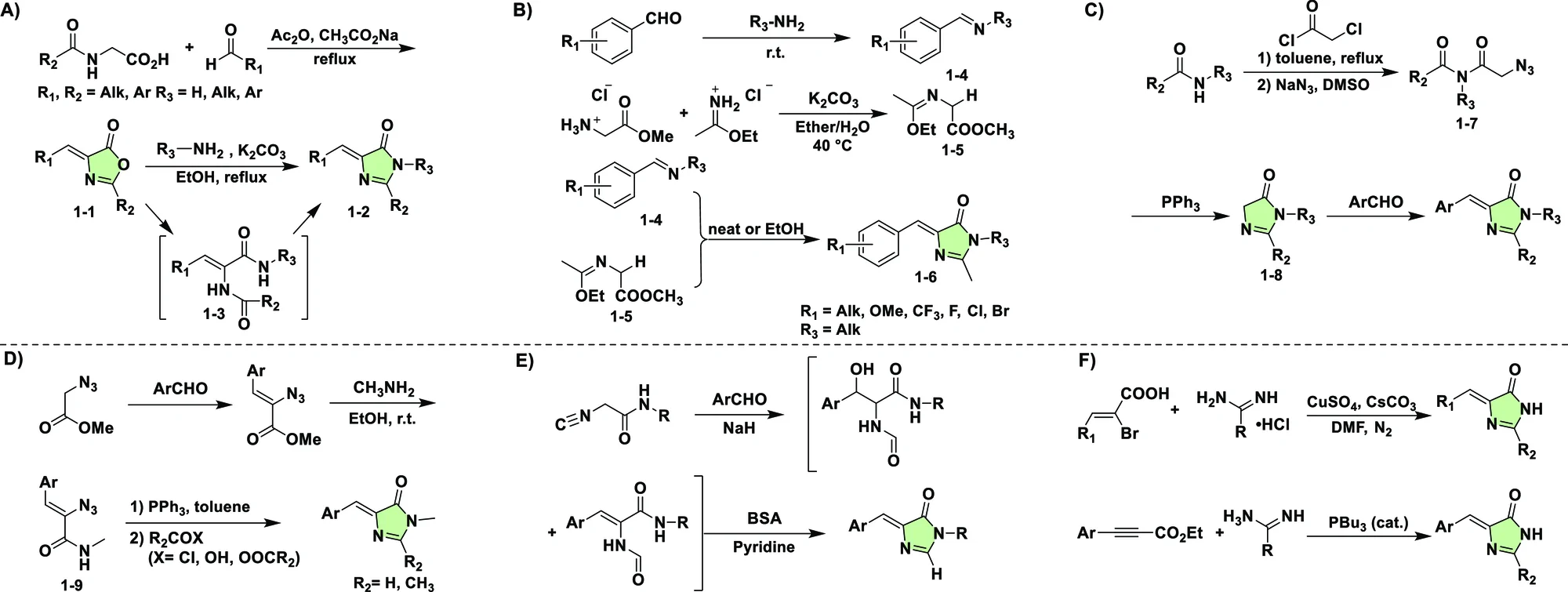
Common Synthetic Strategies for Constructing the GFP Chromophore Core Imidazolinones; BSA, Bistrimethylsilylacetamide

Other classical synthetic routes following well-established organic reactions are also employed, for instance arylideneimine and glycine imidates can be combined to produce **HBDI**s, both readily accessed from common building blocks (**1-4** and **1-5**, Scheme 1B).^42–45^ This 2 + 3 cyclocondensation reaction can be conducted under mild conditions, with the products often readily obtained through simple precipitation without any further purification.^43^ Synthetic limitations in this case include structural variation at C-2, which is limited by suitable and available imidates (only Me shown). Aza–Wittig reactions are also employed (Scheme 1C,D).^41,46,47^ As show in Scheme 1C, azide intermediates **1-7** can be readily prepared from commercial amides and then converted to C-4 unsubstituted oxazolidinones **1-8** by a Staudinger/aza-Wittig-based cyclization, finally functionalized by aldol condensation with an aldehyde to afford the GFP-type chromophore. In a slight reordering of steps, Yampolsky and co-workers have also reported an aza-Wittig-based approach for synthesizing 2H-imidazolones wherein cyclization occurs as the final step (Scheme 1D).^47^ 2H-imidazolones can also be synthesized from isocyanoacetamides in a two-step one-pot process involving condensation with an aldehyde and cyclization using bistrimethylsilylacetamide as an efficient cyclocondensation reagent, as reported by Bischoff et al. (Scheme 1E).^40,48^ Finally, amidines are also common in the synthesis of **HBDI** derivatives, enabling facile access to 2-H imidazolinones (Scheme 1F). These processes can proceed either from the α-halo acrylic acids or yne-esters using either copper-catalyzed^49^ or tributylphosphine-catalyzed^50^ reactions, respectively.

## 3 Photophysical Characteristics of the GFP Chromophore and Its Analogues

As noted earlier, fluorescence enhancement in **HBDI**-based systems fundamentally relies on suppressing torsional motions that drive nonradiative decay (Figure 2A).^27–30,51^ The exact underlying mechanisms remain under active investigation.^51–55^ Figure 2B–D, shows the core chromophore structures of GFP, derived from the **HBDI** scaffold.^56^ Since intramolecular motion provides efficient nonradiative relaxation pathways for the excited-state of the chromophore, inhibiting or modulating such motion offers an effective strategy to tune the Φ and brightness of GFP chromophore analogues. As will be discussed in the next section, these photophysical properties can be, and have been, exploited to develop fluorescent sensors for bioanalytes capable of inhibiting intramolecular motion of GFP chromophores (e.g., ions, DNA, RNA, proteins).

**Figure 2:**
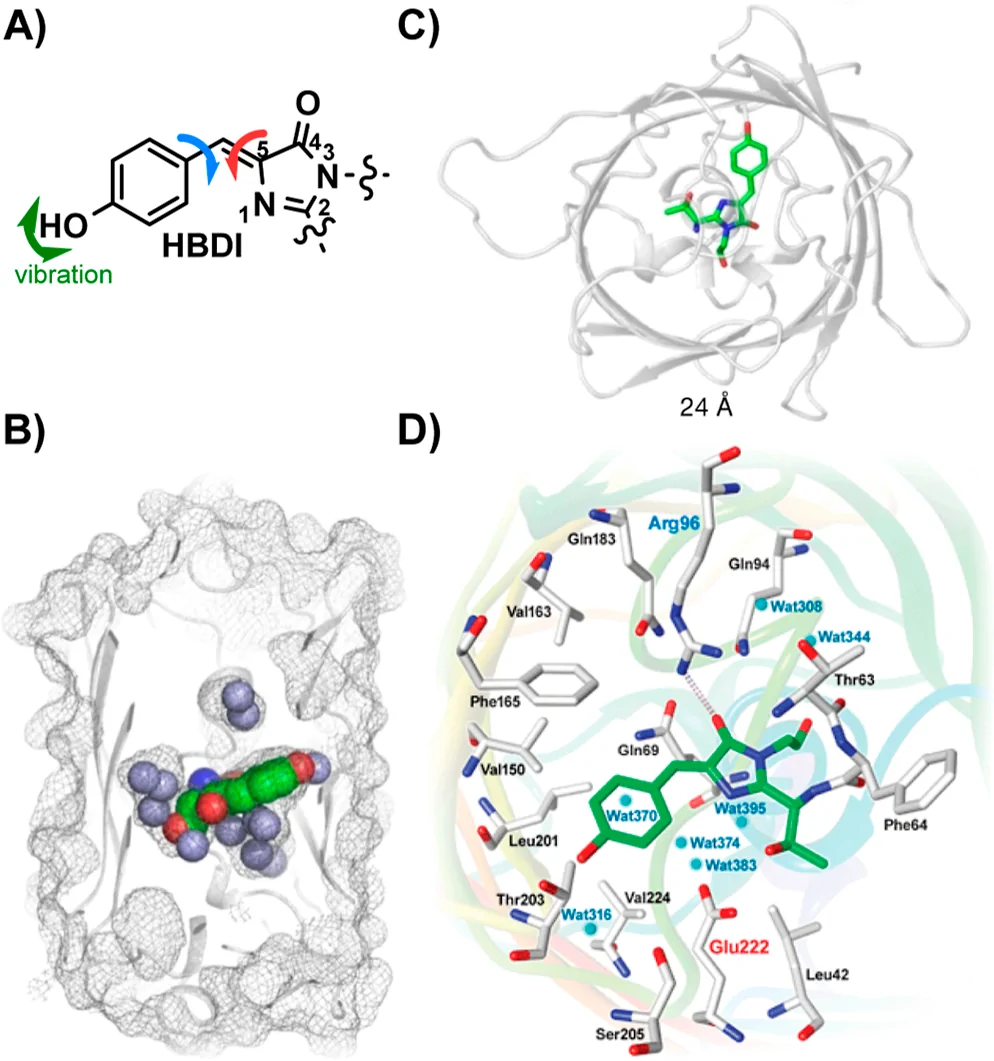
(A) Proposed mechanisms for very weak fluorescence of free GFP chromophore. As the green and blue arrows indicate, free vibration and rotation around the aryl-alkene bond (φ torsion) cause the loss of energy. Isomerization of the alkene (red arrows, τ torsion) also dissipates energy through nonradiative pathways. Atom numbering has been provided for clarity and convenience in the discussions. (B) Surface mesh representation of GFP, based on the X-ray crystal structure (Protein Data Bank ID: 1EMA). (C) Discussions. (B) Surface mesh representation Axial view (along the β-barrel axis) of GFP shown in ribbon representation, with the chromophore located in the central pocket. (D) Chromophore environment, chromophore shown in green (other atoms: carbon in white, nitrogen in blue, oxygen in red, structural water in light blue). (B,C) Adapted with permission from ref 57 copyright 2018 Springer Nature Limited; (D) reproduced with permission from ref 58 Copyright 2009 The Royal Society of Chemistry.

The impact of rigidification has been investigated by Chou et al., who prepared a series of locked *ortho*- and *para*-substituted GFP chromophores designed to modulate emission via structural constraints.^59^ They initially synthesized the dye **3-1** (Figure 3A) which can form a seven-membered ring through intramolecular hydrogen bonding between the *o*‑phenol hydroxyl (donor) and the imidazolinone nitrogen (lone pair, acceptor).^60^ Compared to **3-2** where intramolecular hydrogen bonding cannot occur, its Φ value in water was enhanced nearly 10-fold from 0.9 × 10^–4^ to 9 × 10^–4^,^59^ with the former comparable to the Φ value of the reference **HBDI** compound (1 × 10^–4^).^25^ The formation of a seven-membered ring through intramolecular hydrogen bonding and concomitant rigidification hinders torsion of the GFP chromophore structure to generate an increased Φ. Further locking was achieved in compounds **3-4** and **3-5** where φ rotation around the aryl-alkene bond was constrained by cyclization.^59^ Compared to the unconstrained structure of **3-3** with a Φ value at 2.3 × 10^–3^ in water, **3-5** exhibited a much higher Φ (nearly 40-fold increase to approximately 0.1).^59^ **3-4** on the other hand, featuring alkene restriction only through cyclic functionalization but no H-bonding, exhibited a low Φ value (5.1 × 10^–4^ in water), further suggesting that formation of the seven-membered H-bonding ring is the key contributor, whereas introduction of a cyclic pattern on the benzylidene is a relatively minor contributor. It should be noted that while several analogs were evaluated in water, others were measured in organic solvents. As Φ values are strongly solvent dependent, meaningful quantitative comparison is therefore only appropriate for compounds measured under the same solvent conditions.

**Figure 3:**
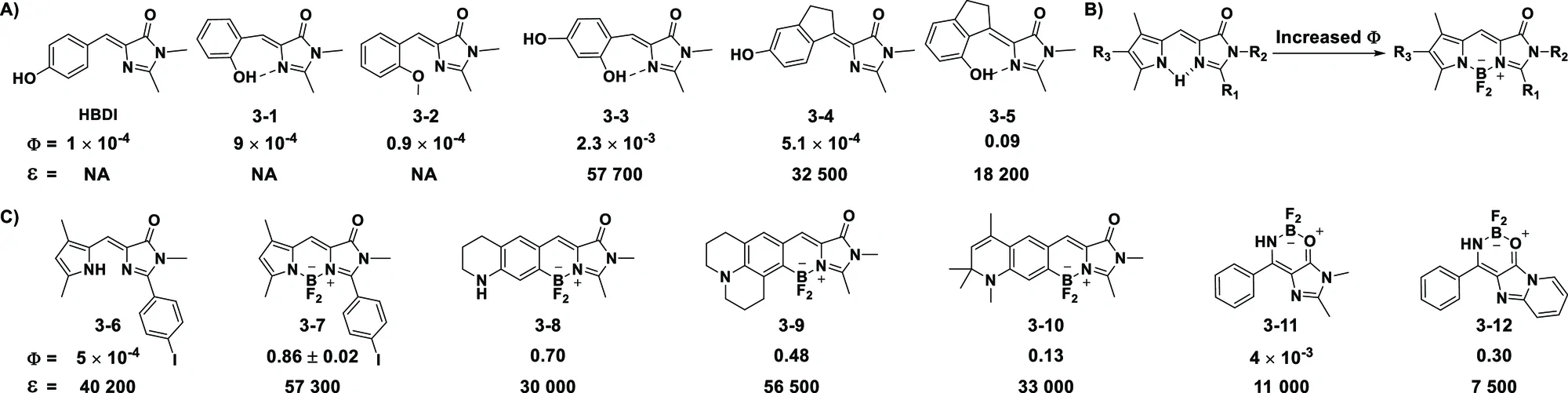
Structures and fluorescence quantum yields (Φ) and extinction coefficients (ε, M cm)^−1^ of different GFP chromophore analogues. (A) **HBDI** and analogues with/without intramolecular hydrogen bonding and/or a cyclic substituent. Measured in benzene (**HBDI**), Water (**3-1**, **3-2**),^60^ Water (**3-3**, **3-4**),^59^ CH_3_CN (**3-5**).^59^ (B) Boron-complexed **HBDI** analogues. (C)­Measured in MeOH (**3-6**, **3-7**),^41^ Water (**3-8**–**3-10**),^61^ Water (**3-11**, **3-12**).^62^ (Caution should be exercised when directly comparing absolute Φ values across different compounds. NA: indicates the listed assay was not conducted).

Given the significant increase in fluorescence caused by rigidification and planarization arising from this hydrogen bonding network, other analogues that achieve a similar structural outcome have been widely explored. For instance, Burgess et al. synthesized a series of BODIPY-like boron-containing complexes, where a pyrrole benzylidene substituent and unsubstituted imidazolinone nitrogen coordinate a central bridging boron atom (Figure 3B).^41^ This modification afforded a remarkable increase in Φ, with a near 2000-fold increase in Φ value on going from nonborate reference compound **3-6** (Φ = 5 × 10^–4^) to difluoroborate **3-7** (Φ = 0.86). Similarly, Baranov, Solntsev and Yampolsky et al. synthesized a series of nonpyrrole analogues with aryl difluoroborates which exhibited a comparable increase in Φ, (e.g., **3-8**–**3-10**, Φ = 0.13–0.70 in water).^,^ ^61^ The third and final series of rigid boron-complexed **HBDI** analogues involved the introduction of a benzylic amine, where the amine nitrogen forms a covalent bond with boron, and the imidazolinone carbonyl oxygen coordinates to the same boron center, forming a six-membered chelate ring (**3-11** and **3-12**).^62^ This was also shown to increase Φ values, in particular for **3-12** where it increased to 0.30 in water.

Of course, modulation of Φ is only one property of interest, with wavelengths of emission and absorption another. This is key to the applicability and adoption of the probes, with longer wavelengths typically favored for reduced scattering and better tissue penetration. Modulation of GFP chromophore analogues can be readily carried out using functional modifications, with the aim of moving away from the intrinsic absorption and emission wavelengths of 370 and 435 nm exhibited by **HBDI** at pH 7.0.^37^ For instance, *ortho*-difluorination of **HBDI** induces a redshift of emission wavelength of approximately 55 nm as seen in analogue **4-1** ^63^ (Figure 4), caused by the increased acidity of the phenol resulting in formation of the phenolate in solution.^34^ While, addition of a trifluoromethyl substituent at the imidazolinone C-2 position in **4-2** induces a ca. 40 nm red-shift in maximum excitation emission compared to **4-1**.^63^

**Figure 4:**
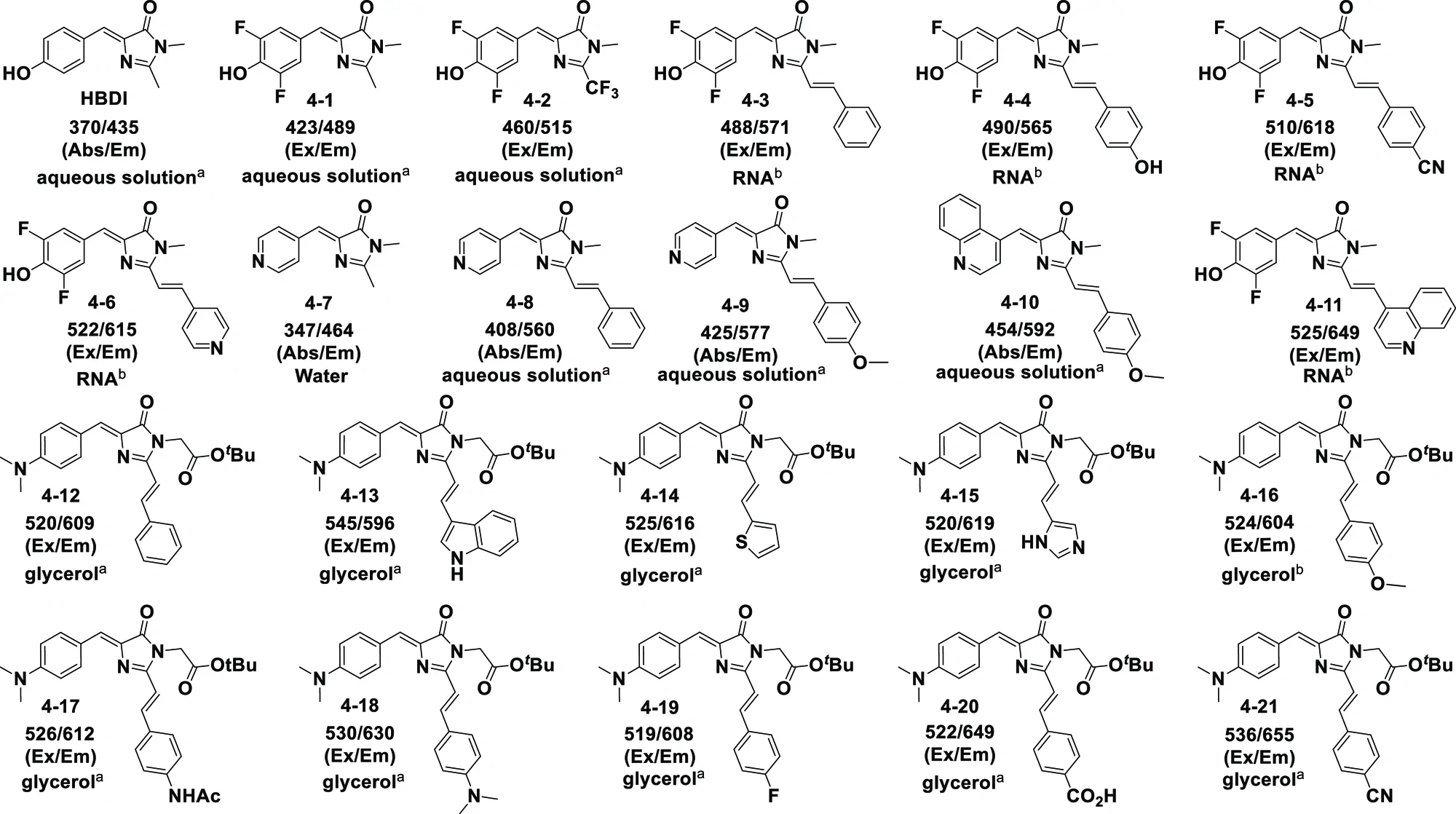
Overview of selected structurally modified GFP chromophore analogues and their different excitation and emission wavelengths (^a.b^ the wavelengths of maximal excitation and emission were measured in either aqueous solution^a^ or during interaction with RNA^b^ in buffer).

As expected, extending the conjugated π-system also induces significant red-shift of the maximum absorption and emission wavelengths of **HBDI** analogues. For instance, compounds **4-3**–**4-6** exhibited a remarkable red-shift in both excitation and emission spectra thanks to simple addition of styryl-based motifs (Figure 4).^36,64,65^ It is important to note that the influence of electron-withdrawing groups on absorption and emission wavelength is nuanced and context-dependent. Both the direction and magnitude of the spectral shift depend on the position of the substituent along the conjugated π-system, and whether it engages primarily through inductive (-I) or mesomeric (-M, resonance) effects (or a balance of both). For instance, an electron-withdrawing substituent placed at a position that allows conjugation with the acceptor segment of a push–pull-type chromophore will enable resonance stabilization of the LUMO, narrowing the HOMO–LUMO gap, resulting in a red-shift, as observed for compound **4-5**. In contrast, substitution at other positions, or substituents dominated by inductive effects, will lead to different, and in some cases opposing, spectral response. Accordingly, rational wavelength tuning requires consideration of the specific electronic structure and substitution patterns of the chromophore. Modifications to the benzylidene are also effective, for instance introduction of a pyridine group to cause a bathochromic shift in emission wavelength. This is seen in compound **4-7**, which exhibited a maximum emission wavelength of 464 nm, 29 nm longer than the maximum emission wavelength of **HBDI**.^66^ Both approaches can be combined to generate an enhanced red-shift in absorption/emission wavelengths, as seen in **4-8** and **4-9** with emission wavelengths in the 560–577 nm range (Figure 4).^66^ Additional extension of conjugation through introduction of quinolines on to the imidazolone also leads to longer wavelengths of absorption and emission (**4-10**,^67^ and **4-11** ^36^).

Along a similar vein, the effect of π-conjugation extension at the C-2 position has been explored through the introduction of heterocycles (**4-12**–**4-15**).^68,69^ Interestingly, *para*-substituent modification of the unsubstituted styrene-bearing analog **4-12** with electron-donating and electron-withdrawing groups produced nearly identical absorption maxima with minor variations in emission wavelength (**4-16**–**4-17** and **4-19**, Figure 4).^68^ In contrast, compounds **4-20** and **4-21** containing electron-withdrawing groups exhibited significantly red-shifted emissions at 649 nm 655 nm, respectively.

The photophysical properties of the GFP chromophore are enhanced by rigidification, which suppresses nonradiative decay. To mimic this, synthetic strategies employ conformational locking, steric hindrance, or electronic modulation to rationally tune absorption and emission.

## 4 Applications of GFP Chromophore-based Probes in Sensing and Imaging

### 4.1 Nucleic Acid Sensing and Nucleic-Acid-Based Fluorescence Reporting

The unique environmental sensitivity of GFP-derived chromophores has been adapted for nucleic acid research, primarily through two distinct strategies that go beyond traditional staining approaches. One strategy involves their covalent incorporation into nucleic acids, where they function as environmentally sensitive fluorescent reporters or labels. In this context, the fluorophore does not selectively bind to the nucleic acid itself but is tethered, and its emission, which is often weak in the free state, can be markedly enhanced in response to changes in the local microenvironment, such as upon binding of the labeled nucleic acid to a protein target. The second, and perhaps more recognized, strategy exploits in vitro RNA aptamers that bind specific GFP chromophore analogues with high affinity and selectivity. In these systems, the aptamer itself provides a rigid protein-mimetic pocket that restricts chromophore motion, resulting in a pronounced fluorescence turn-on. This represents a genetically encodable recognition system based on an in vitro selected RNA aptamer, in which selectivity arises from the engineered RNA–chromophore complex rather than from the intrinsic properties of the chromophore alone. Thus, GFP-derived chromophores are used either as covalently tethered, environment-sensitive reporters or as noncovalent ligands for engineered RNA aptamers that undergo fluorescence activation upon binding.

For instance, GFP-like chromophores, 3,5-bis­(methoxy)-4-hydroxybenzylideneimidazolinone (MBI) and 3,5-difluoro-4-hydroxybenzylideneimidazolinone (FBI)^70^ were successfully conjugated to dCTP via a propargyl linker, affording the fluorescently labeled nucleotides dC^MBI^TP and dC^FBI^TP. These labeled nucleotides were efficiently incorporated into DNA through polymerase-catalyzed primer extension reactions. The resulting fluorescently labeled DNA exhibited low emission in the free state. However, upon binding of the tumor suppressor protein p53 through the recognition sequence within the labeled dsDNA, the fluorescence intensity increased 2.3-fold for MBI (at 484 nm) and 2.1-fold for FBI (at 496 nm), consistent with the more hindered rotation of the fluorophore due to interaction with the bound protein. Furthermore, dC^FBI^TP was employed in time-resolved experiments to monitor the incorporation of a single nucleotide, followed by primer extension catalyzed by Vent (exo-) polymerase (Figure 5A).

**Figure 5:**
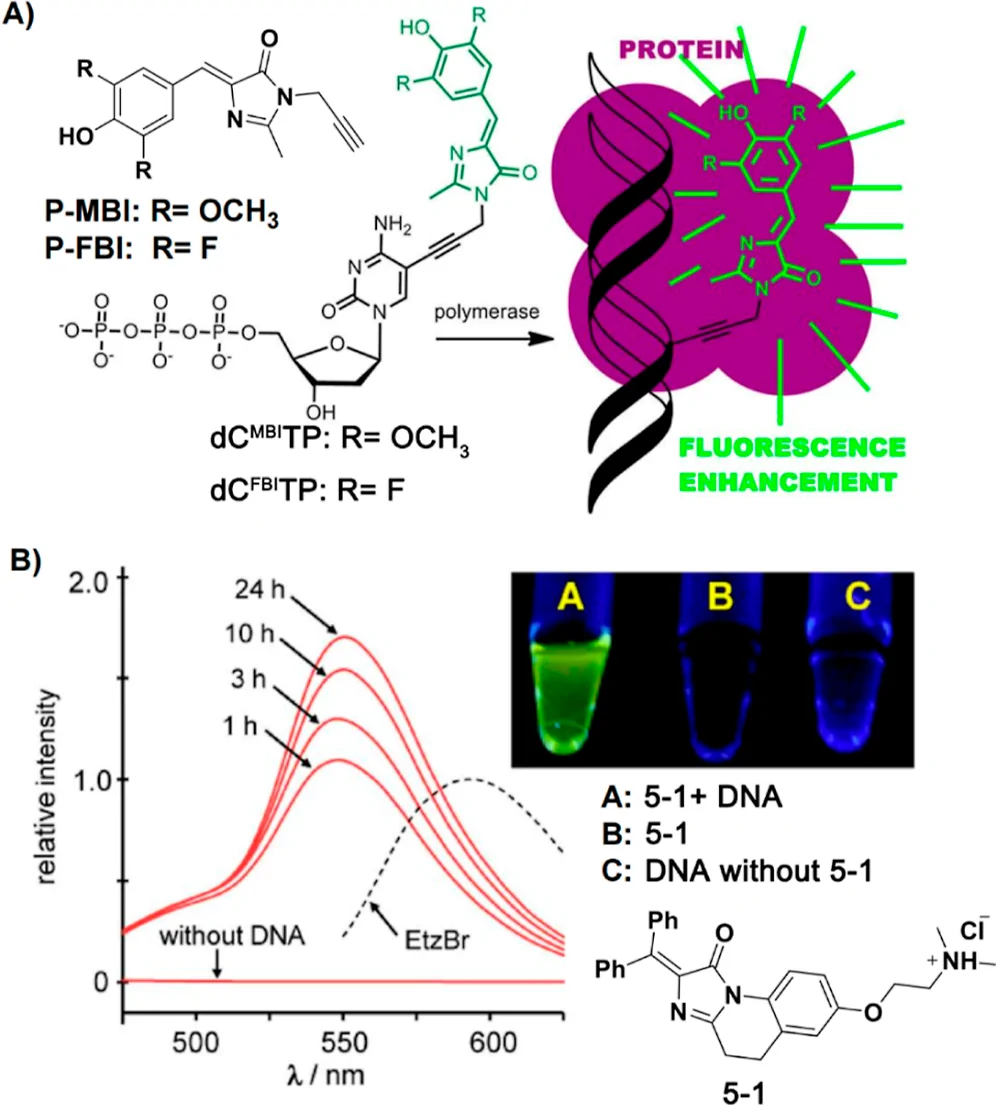
(A) P-MBI, P-FBI, labeled nucleotides (dC^MBI^TP and dC^FBI^TP), and the process of incorporating labeled nucleotides into DNA during primer extension, along with the fluorescence output under polymerase catalysis. (B) Fluorescent spectra and image of **5-1** in the presence of double-stranded DNA (0.01% of 5–1 (2.1 × 10^–4^ M) and 0.025% of DNA in water, λ_ex_ = 327 nm). The same concentration of ethidium bromide was used as a standard for the relative intensity. Panel A adapted with permission from ref 70 Copyright 2012 American Chemical Society; Panel B adapted with permission from ref 71 Copyright 2012 American Chemical Society.

Another DNA-sensing system is that of Miyashita and co-workers, who developed compound **5-1** (Figure 5B) that could detect double-stranded DNA with at least a 500-fold increase in fluorescence intensity, exhibiting much better performance relative to ethidium bromide (only ca. 10-fold increase in fluorescence intensity).^71^ While ethidium bromide has historically served as a common DNA stain, it is now recognized as mutagenic and suboptimal for sensitive applications, creating a need for improved DNA sensing motifs.

Also common is the use of GFP-based chromophores and their analogues in RNA sensing and live cell imaging. In 2011, Jaffrey and colleagues screened a variety of RNA sequences to study their binding to **HBDI** and analogues.^34^ RNA 13-2 was found to selectively activate the fluorescence of the dimethoxy analogue **6-1** (Figure 6A). Several other specific RNA sequences were then identified that enabled specific fluorescent activation of other **HBDI** analogues such as **4-1** and **3-1** (same structures as **4-1** [Figure 4], and **3-1** [Figure 3], respectively), as well as differential response with **6-1** (Figure 6B,C). Notably, RNA 24-2 selectively binds **4-1** to produce bright green fluorescence with an enhanced Φ of 0.72, surpassing that of the industry-standard EGFP (Φ = 0.60).^72^ Additionally, the RNA-24-2**-4-1** complex experienced negligible photobleaching, with its favorable properties earning it the nickname “Spinach” due to its green fluorescence emission.

**Figure 6:**
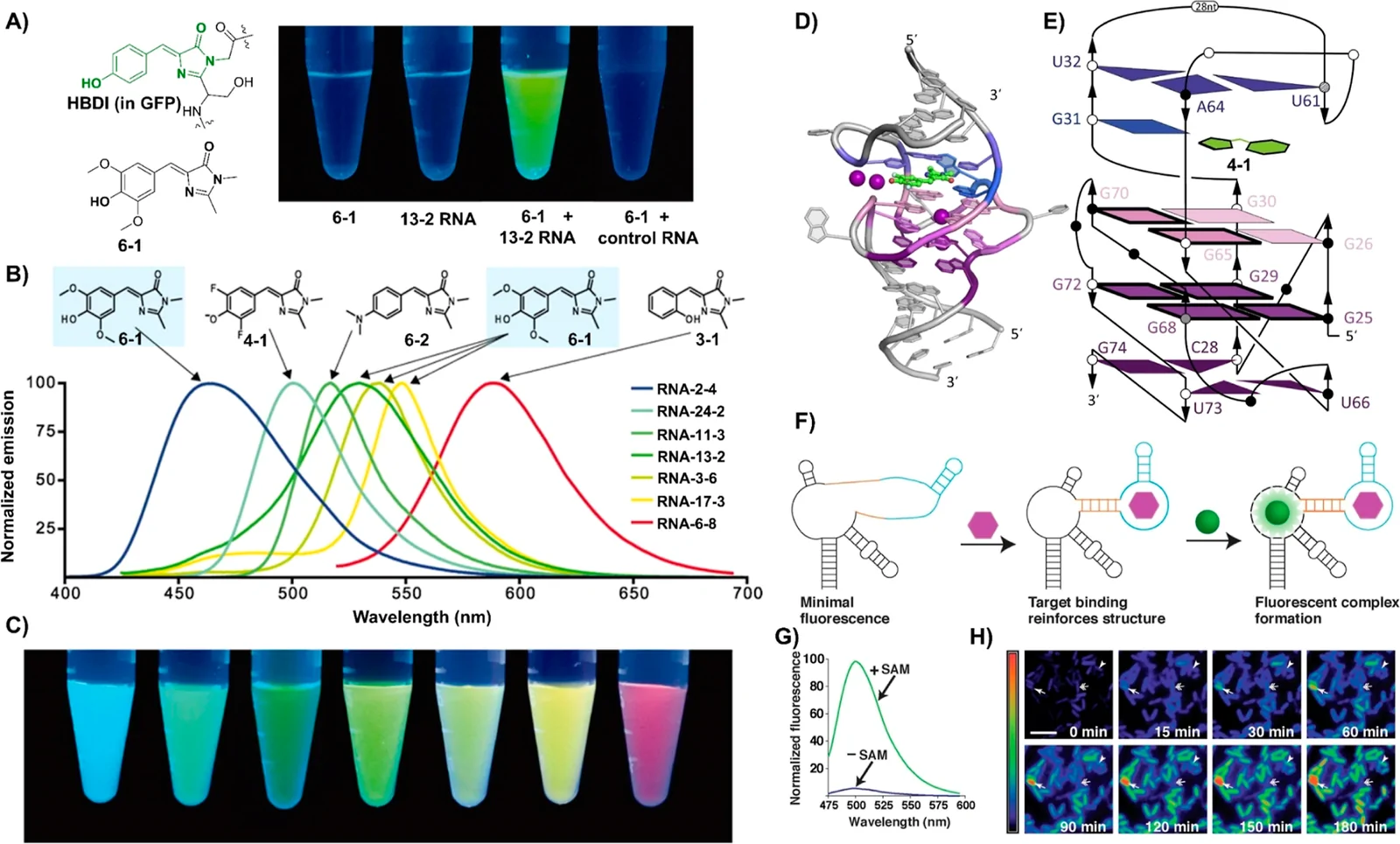
(A) Structures of **HBDI** (green) in GFP, and **6-1**. Image demonstrating that RNA 13-2 enhances the fluorescence of **6-1**. (B) Fluorescence spectra of RNA-fluorophore complexes. Spectra were collected in the presence of excess fluorophore at pH 7.4 for RNAs binding to **6-1** (RNA-2-4, RNA-13-2, RNA-3-6, and RNA-17-3), **4-1** (RNA-24-2), **6-2** (RNA-11-3), and **3-1** (6-8). Spectra for each complex are normalized to their respective emission peaks. (C) RNA-fluorophore complexes were illuminated with ultraviolet light (365 nm) and photographed. Left to right: RNA-2-4, RNA-24-2, RNA-11-3, RNA-13-2, RNA-3-6, RNA-17-3, RNA-6-8, and fluorophores as indicated above. (D) Structural core of the Spinach aptamer complexed with its fluorophore **4-1** (shown as green, ball-and-stick representation) reveals metal ions (purple spheres), as resolved in the crystal structure (Protein Data Bank ID: 4TS0). (E) A schematic illustration of the Spinach aptamer binding pocket that highlights base connectivity and stereochemistry. The base colors correspond to those in panel (D), with bold outlines indicating *anti* nucleoside conformations and thin outlines indicating syn. Ribose sugar puckers are annotated open circles for 3′-endo, black circles for 2′-endo, and G68 adopts a 4′-endo conformation.^73,74^ (F) Illustration of sensing mechanism of Spinach-**4-1** based RNA sensor for detecting small molecules. (G) Emission spectra of the Spinach-**4-1**-based SAM sensor in the absence and presence of SAM. The fluorophore is **4-1**, and the RNA component is an engineered chimeric SAM-sensing RNA composed of a SAM-binding aptamer, a transducer stem, and the Spinach fluorescent aptamer. SAM binding promotes folding of the RNA sensor and activates **4-1** fluorescence. (H) Time-lapse fluorescence imaging of dynamic SAM changes in individual *E. coli* cells expressing the SAM sensor RNA after methionine addition. Images were acquired at the indicated time points (0 to 180 min). The pseudocolor scale represents the fold increase in fluorescence intensity at each time point relative to the starting point (*t* = 0 min), ranging from 0 (blue) to 11.2-fold (red). Arrows highlight representative cellular responses: a cell with sustained high SAM accumulation (arrow), a cell with a slow increase (arrowhead), and a cell exhibiting a transient increase followed by a decrease (double arrow). Scale bar, 5 μm. Panels A and B adapted, and Panel C reproduced with permission from ref 34 Copyright 2011 American Association for the Advancement of Science. Panels D reproduced and E adapted with permission from ref 74 copyright 2017 Elsevier Ltd. Panels F, G, H reproduced with permission from ref 75 Copyright 2012 American Association for the Advancement of Science.

The photophysical properties of Spinach-**4-1** complex have enabled its use in live-cell imaging applications, including the visualization of Spinach-tagged RNAs and Spinach-based RNA sensors. The cocrystal structure of Spinach bound to **4-1** revealed that the RNA aptamer activates fluorescence by immobilizing the fluorophore within a specific binding pocket, where it is sandwiched between a G-quadruplex and a base triple (Figure 6D,E).^73,74^ This binding mode functionally resembles the conformational restriction of the **HBDI** chromophore in GFP discussed above. As in other fluorogenic RNAs,^73^ the G-quadruplex plays a key role in activating the fluorescence of Spinach-**4-1**, which provides a new strategy of designing GFP chromophore analogue-RNA complexes for fluorescence sensing and imaging.

In addition to RNA sensing, Spinach-**4-1** could be used to construct RNA-based sensors for detecting small molecules by linking the small molecule–binding aptamers to Spinach. The unstructured small molecule–binding aptamers could be folded after selectively binding to the target small molecules, stabilizing the stem between Spinach and the small molecule–binding aptamers to produce fluorescence (Figure 6F).^75^ These RNA-based sensors have been used for detecting *S*-adenosylmethionine (SAM, Figure 6G), as well as imaging the dynamic changes of SAM in *E. coli* (Figure 6H).

Spinach, however, has certain limitations such as thermal instability and a propensity for misfolding in live cell imaging. Jaffrey, Ferré-D’Amaré and colleagues therefore developed Spinach2,^76^ “baby Spinach”,^73^ and “Broccoli”^77^ to address some of these issues.^78^ For example, Spinach2-**4-1** exhibited improved stability in live cell fluorescence imaging of CGG repeat-containing RNA compared to Spinach-**4-1** (Figure 7A).^76^ Furthermore, Spinach2 enabled the expansion of the fluorescence window through modification of **4-1** to produce **7-1** and **7-2**, both of which exhibited longer excitation and emission wavelengths. These red-shifted fluorophores render the system more suitable for use with existing GFP and yellow fluorescent protein filter cubes, respectively, thereby increasing the acceptability and potential for uptake by the biosciences community. Additionally, **7-1** exhibits both brighter fluorescence and lower background signals than **4-1** in the presence of Spinach2 when used for live cell imaging.^63^

**Figure 7:**
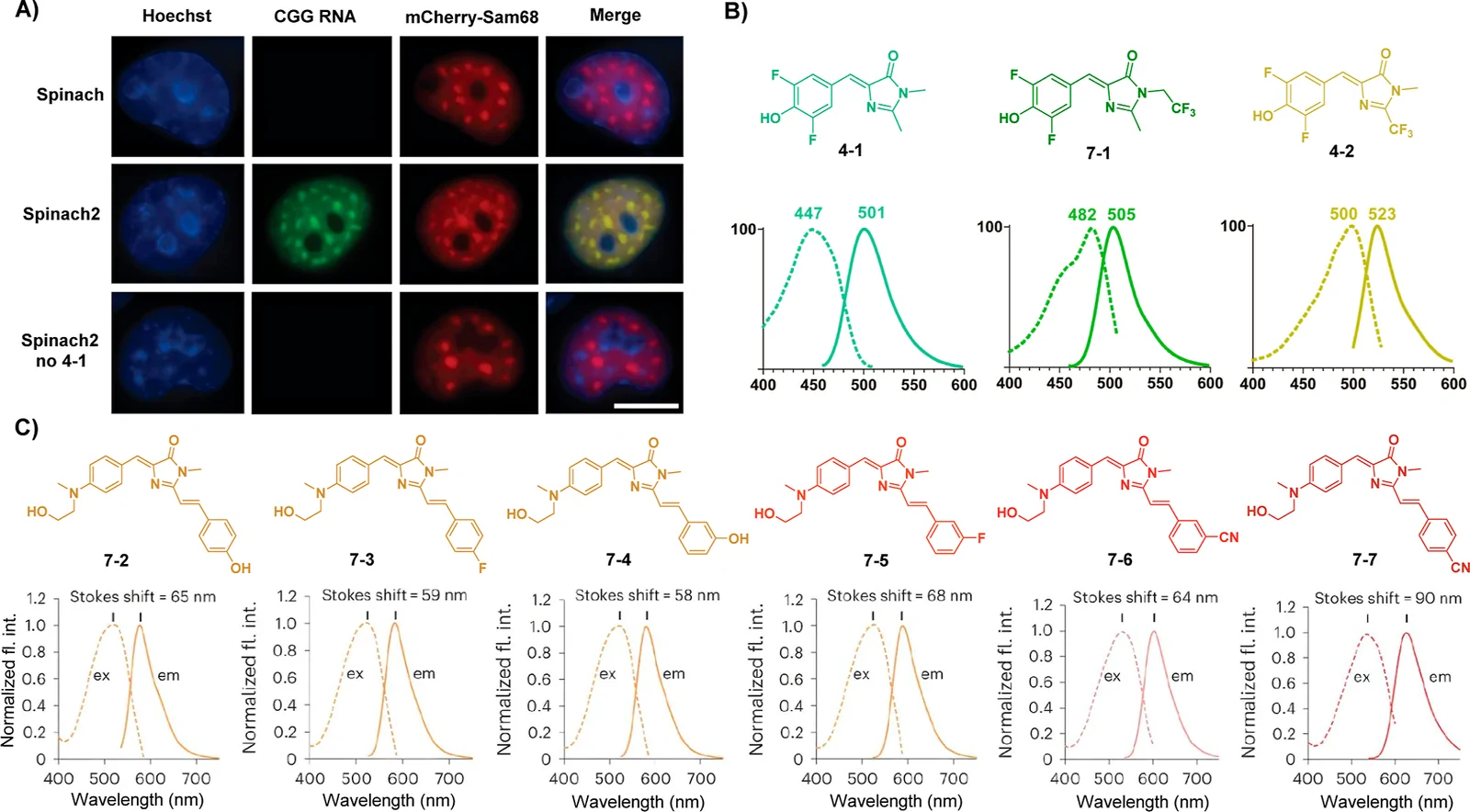
(A) Fluorescence imaging of Spinach- or Spinach2-tagged (CGG)_60_ RNA in live cells. COS-7 cells were cotransfected with plasmids encoding (CGG)_60_–Spinach or (CGG)_60_–Spinach2 RNA along with mCherry–hSam68 fusion protein, and imaged in the presence of **4-1**. As a control, cells expressing (CGG)_60_–Spinach, (CGG)_60_–Spinach2, and mCherry–hSam68 were also imaged without **4-1**. Nuclei were counterstained with Hoechst. Scale bar: 10 μm. (B) Structures of **4-1**, **7-1** and **7-2**, and their excitation (dotted line) and emission (solid line) spectra in the presence of Spinach2. (C) **NBSI** series and their Stokes shifts, excitation (dashed line) and emission (solid line) spectra in the presence of Clivias. Panel A adapted with permission from ref 76 Copyright 2013 Springer Nature America, Inc. Panel B adapted with permission from ref 63 Copyright 2014 American Chemical Society. Panel C adapted with permission from ref 64 Copyright 2023 the authors, published by Springer Nature.

In 2023, Yang, Zhu, Ren, Chen and colleagues developed a new series of GFP chromophore analogues (**NBSI** dyes) by introducing various donor and acceptor substituents and extending the π-conjugated system (Figure 7C). To activate these dyes in cells, they evolved a new small monomeric RNA aptamer named Clivias that specifically binds and lights up **NBSIs**.^64^ These aptamer–fluorophore complexes exhibited large Stokes shifts, with **7-7** for instance displaying a 90 nm separation between its excitation and emission maxima. However, due to the broad nature of both spectra, some overlap between the excitation and emission profiles remains.

Small-molecule dyes are generally less bulky than fluorescent proteins and can therefore reduce steric and functional interference with the target biomolecule. However, when they are used in combination with RNA aptamers, the aptamer sequence must be genetically encoded into the target RNA or sensor RNA, which may offset some of this size-related advantage over fluorescent protein fusions. Therefore, the benefit of reduced interference is most evident when small-molecule dyes are used as stand-alone probes without aptamer engineering.

### 4.2 Protein Sensing and Imaging

Proteins are another essential component of cells, regulating and enabling many biological processes; disruption of their function can therefore contribute to a wide range of diseases and disorders. For instance, protein misfolding and aggregation processes are closely associated with various human diseases, particularly neurodegenerative conditions such as Alzheimer’s disease, Parkinson’s disease, Huntington’s disease, and amyotrophic lateral sclerosis.^79^ An example application of GFP chromophore analogues in this context is that reported by Zhang and Liu et al., who developed a series of **HBDI** analogues (**8-1** and **8-2**, Figure 8A) that could be used for sensing protein aggregation.^69^ In this instance, protein misfolding or aggregation inhibits the intramolecular motion of **8-1**, leading to enhanced fluorescence in rigid environments such as protein aggregates. While compound **8-1** is mainly used for in vitro aggregation assays, its derivative, probe **8-2** (Figure 8B) incorporates a HaloTag-reactive ligand that enables bioorthogonal labeling of a protein-of-interest in live cells. When probe **8-2** was conjugated to a Halo-Tag fusion of the aggregation-prone Huntingtin exon 1 protein with 97 polyglutamine repeats (Htt-Q97-Halo) in HEK293T cells, strong punctate fluorescence was observed, indicating protein aggregation. In contrast, cells expressing nonaggregating Htt-Q0-Halo showed no fluorescence after labeling with **8-2** (Figure 8B­(c)). A similar trend was observed in cell lysates (Figure 8B­(d)). These results demonstrate the efficiency and specificity of the **8-2**/Halo-Tag-based approach for detecting protein aggregation in live cells.

**Figure 8:**
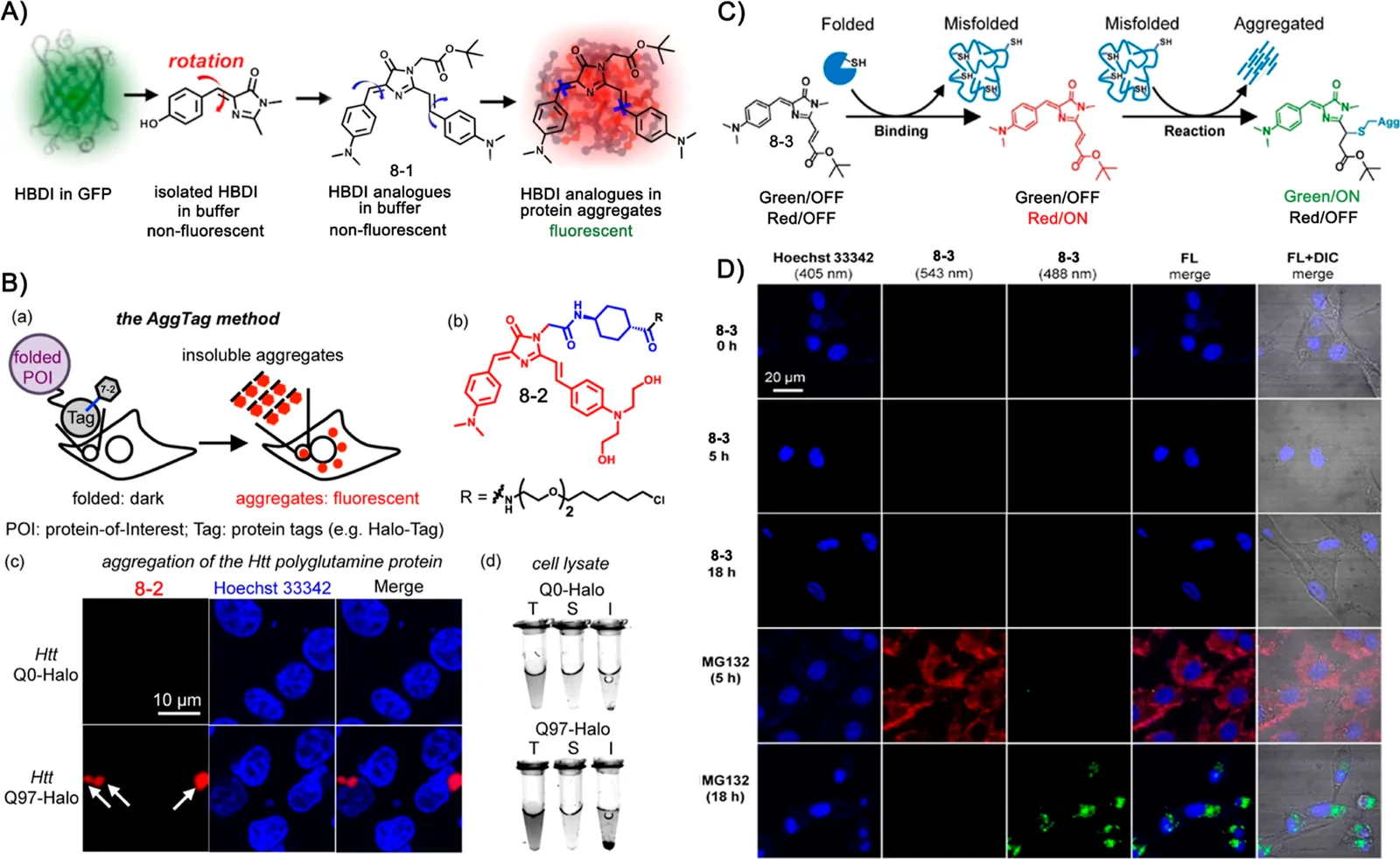
(A) Design principle and mechanism for **HBDI** analogues **8-1** for detecting protein aggregates. (B) Probe **8-2** with Halo-Tag ligand for the detection of specific proteins in live cells. (a) Design strategy; (b) Structure of probe **8-2**; (c) Probe **8-2** for imaging Huntingtin exon 1 protein (Htt) with 97 polyglutamine repeat (Htt-Q97) with Halo-Tag protein (Htt-Q97-Halo); (d) Fluorescence based images of cell lysate from cells expressing Htt-Q0-Halo or Htt-Q97-Halo labeled by **8-2** after fractionation; T, total lysate; S, supernatant; I, insoluble fraction. (C) Proposed sensing mechanism of probe **8-3** for detecting protein misfolding and aggregates. (D) Fluorescence images of aggregated proteome in stressed live U251 cells with **8-3**. Cells were stressed by MG132 (proteasome inhibitor) to induce protein degradation and aggregation. Panels A and B adapted with permission from ref 69 Copyright 2018 American Chemical Society. Panels C and D adapted with permission from ref 80 Copyright 2021 Wiley-VCH GmbH.

Liu, Li and Zhang et al. then developed **HBDI** analogues with different photophysical and viscosity sensitivity properties to distinguish unfolded protein oligomers from insoluble aggregates in live cells.^68^ In 2021, Liu and colleagues developed a new probe **8-3** for the selective detection of aggregates via conjugate addition to its α,β-unsaturated ester functionality (Figure 8C).^80^ In a misfolded protein, **8-3** fluoresces red. As misfolding progresses insoluble aggregates form, proximity and exposure combine to allow reactive thiols (e.g., cysteine residues) to form covalent bonds with **8-3**, switching the fluorescence emission to green by reducing the size of the π-conjugated system (blue-shift, Figure 8C). This was confirmed in stressed live cells. Initially, the cells exhibited negligible fluorescence after incubation with **8-3**, even after extended incubation times (18 h). Then a strong red fluorescence signal was observed after the cells were treated with MG132 (proteosome inhibitor) for 5 h, indicating the formation of misfolded proteins. Further incubation with MG312 up to 18 h led to the expected emission change from red to green following the formation of aggregates from the accumulation of misfolded proteins (Figure 8D).^80^

The effectiveness of **HBDI**-based probes in reporting protein misfolding and aggregation arises from their sensitivity to physicochemical changes associated with protein conformational collapse, rather than from selectivity toward a specific protein. From research conducted in both refs 70 and 81 , probe activation is governed by state-dependent mechanisms that respond to protein misfolding and aggregation within the investigated model systems. Accordingly, these probes operate through two complementary modes that are dependent on protein conformation rather than sequence: (1) genetically encoded localization strategies, exemplified by probes such as **8-2** that are delivered to proteins of interest via bioorthogonal tagging systems (e.g., HaloTag), enabling controlled positioning without conferring intrinsic protein selectivity; and (2) conditional chemical reactivity, as demonstrated by probe **8-3**, which exploits aggregation-associated exposure of reactive thiols (e.g., cysteine residues) to enable covalent labeling.

For turn-on probes such as **8-1** and **8-2**, fluorescence enhancement arises from restriction of intramolecular motion within the rigid and crowded microenvironment of protein aggregates, analogous to the conformational locking provided by the GFP β-barrel. Probe **8-3** introduces an additional chemical dimension: its α,β-unsaturated ester functions as a Michael acceptor, enabling a covalent reaction with exposed thiols upon aggregate formation. This reaction alters the π-conjugation of the chromophore and shifts the emission from red to green, providing a spectrally distinct record of later aggregation states relative to earlier misfolded forms. Together, these studies demonstrate how the **HBDI** core can support sensitive, state-dependent reporting of protein misfolding and aggregation without invoking protein-specific recognition.

Another important class of GFP chromophore analogues for the study of proteins are the 4-hydroxybenzylidene-rhodanines (**HBRs**) (Figure 9A). **HBRs** are structurally very similar to **HBDIs**, with the addition of two sulfur atoms to the imidazolinone functionality—one to replace the imidazolinone ring nitrogen atom, and the other at the C-2 position to afford a thiocarbonyl. Free **HBR** and its derivatives are nonfluorescent in solution, and their fluorescence is activated on binding to small monomeric proteins termed fluorescence-activating and absorption-shifting tags (FASTs).^81,82^ As with **HBDIs**, intramolecular motion of **HBRs** leads to nonradiative relaxation, and hence very weak fluorescence. The protein cavity of FASTs restrict this intramolecular motion to stabilize the anionic fluorescence state of **HBR** and its derivatives (Figure 9A),^81,82^ enhancing the fluorescence signal relative to unbound **HBR** for a “turn-on” response. This binding is specific and reversible, which enables the dynamic monitoring of specific FAST-tagged protein-of-interests in live cells.^83^ In 2021, the Gautier group optimized their FAST sequence to generate a promiscuous FAST variant named pFAST, which exhibited high binding affinity and fluorescence intensity for a number of **HBR** analogues. A series of **HBR** analogues with spectral properties spanning the entire visible spectrum was developed (Figure 9B) and applied for fluorescence imaging in various cellular localizations in mammalian cells and cultured neurons (Figure S1A).^84^ Another important advance in FAST/**HBR** technology was the development of splitFAST for real-time monitoring and detection of both the formation and dissociation of protein assemblies in the presence of **HBR** analogues (Figure S1B), which was demonstrated in HEK293T cells coexpressing FK506-binding protein (FKBP)-CFASTn (n = 10 or 11) and FKBP-rapamycin-binding domain of the mammalian target of rapamycin (FRB)-NFAST.^85,86^

**Figure 9:**
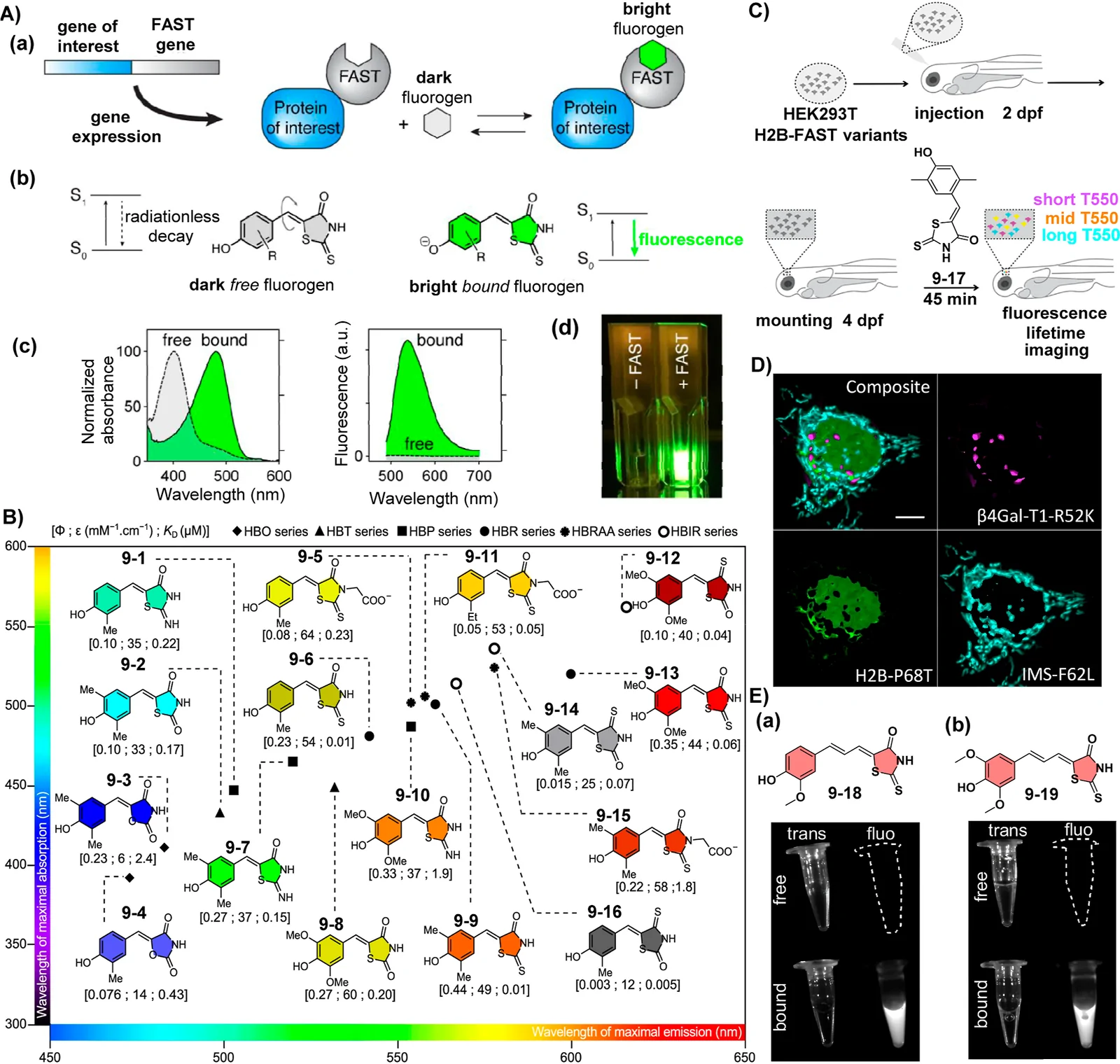
(A) Design principle of fluorescence-activating and absorption shifting tags (FASTs) for protein labeling. (a) Design strategy. Typical FAST is a small monomeric protein of 14 kDa that can be used to monitor gene expression and protein localization in live cells and organisms; (b) Proposed mechanism of fluorescence activation on binding of **HBR** chromophores to FAST; (c) Normalized absorption and emission spectra of **HBRs** before and after FAST binding; (d) Fluorescence images of 4-hydroxy-3-methylbenzylidene rhodanine (**HMBR**, **9-6**) upon binding to recombinant FAST. (B) Structures of a series of **HBR** analogues (including **HBO**, **HBT**, **HBP**, **HBR**, **HBIR**, and **HBRAA** series). In the scheme, each chromophore is placed along the spectrum according to the peak emission and peak absorption wavelengths of its pFAST complex, while the brackets beneath every structure list the complex’s ϕ, molar extinction coefficient at the absorption maximum (ε), and equilibrium dissociation constant (K_D_). (C) HEK293T cells expressing H2B-shortT-FAST, H2B-midT-FAST, or H2B-longT-FAST were pooled 24 h post-transfection and injected near the brain of 2 dpf zebrafish larvae. Fluorescence lifetime imaging was performed at 4 dpf after 45 min incubation with **9-17**. (D) Color assignment based on τ_i_ ranges specific to R52K, P68T, and F62L complexes with **9-17**, showing the composite image and individual localizations. Scale bars: 10 μm. (E) Structures of **9-18** (a, top line) and **9-19** (b, top line). Far-red fluorescence images of free and nirFAST-bound forms of **9-18** (a, bottom line) and **9-19** (b, bottom line). Panel A adapted with permission from ref 81 Copyright 2022 American Chemical Society. Adapted with permission from the Wiley-VCH GmbH (Panel C, 2024, ref 87), and Springer Nature (Panels B, D, E, 2019, 2024, 2025, refs 84, 88, and 89).

To increase multiplexing capacity within a single spectral channel, recent studies have engineered FAST variants that exhibit distinct fluorescence lifetimes, enabling the discrimination of multiple FAST-tagged targets using fluorescence lifetime imaging microscopy (Figures 9C,^87^D^88^). Concurrently, the FAST platform has been extended into the near-infrared (NIR) window with the development of nirFAST, a small chemogenetic reporter that binds cell-permeant synthetic fluorogens. In particular, nirFAST binds **9-19** to form nirFAST715, a bright and photostable NIR complex with an emission at 715 nm (Figures 9E and S2). This red-shifted spectral profile provides advantages for live-cell and in vivo imaging, including reduced tissue autofluorescence and improved imaging contrast. In addition, the brightness and photostability of nirFAST-derived red/far-red assemblies enabled compatibility with STED nanoscopy, enabling subdiffraction imaging of cellular structures such as microtubules.^89^ Furthermore, the orthogonality between nirFAST and pFAST has been leveraged to create a green-NIR cell cycle indicator, demonstrating the versatility of these chemogenetic tags for advanced live-cell and in vivo applications.

### 4.3 Detection of Ions and Live Cell Imaging

As touched on already with the BODIPY-type **HBDI** analogues (see Section 3 & Figure 3) it is possible to employ one of the imidazolinone nitrogens as a coordinating Lewis base, a feature than can be exploited for the detection of analytes, in particular cationic ones. Baldridge and colleagues have shown this with the **HBDI** analogue **10-1** (Figure 10A), substituting the *p*-phenol with a 2-pyridyl motif.^90^ Using chelation between the pyridine and imidazolinone nitrogen lone pairs **10-1** selectively binds Zn­(II) (Zn^2+^) and Cd­(II) (Cd^2+^) cations to elicit a “turn on’’ fluorescence response. In 2013, Xu and Yang et al. synthesized three new fluorescent probes for the selective detection of Zn^2+^ (**10-3** and **10-4** in Figure 10A).^91^ **10-2** (Figure 10A) contains one imidazole–imidazolinone unit, whereas Di-IMMPIs dimerizes two such units, giving four potential N-donors for Zn^2+^ chelation. On Zn^2+^ binding the Φ jumps from <0.002 to 0.352, 0.451 and 0.194, respectively, for **10-2**, **10-3** and **10-4**. **10-3** shows the strongest affinity (K_d_ < 0.03 mM vs 0.10 mM for **10-4**), likely because the flexible butyl linker imposes less steric strain during chelation. Along the same design principles, Long et al.^92^ developed a GFP chromophore-based fluorescent probe **10-5** (Figure 10B). In this molecule, a bidentate 1,10-phenanthroline core (*N*,*N* donor) is fused to two imidazolinone units, generating an extended π-system. The resulting conjugation and increased rigidity generates strong fluorescence and creates a relatively rigid cavity with four nitrogen coordination sites for Zn^2+^. Together, these features provide **10-5** with outstanding fluorescence turn-on response and high selectivity for Zn^2+^(right-hand side, Figure 10B). Further fluorescence imaging in live HeLa cells confirmed the potential use of **10-5** in tracing Zn^2+^ in biological processes (inset, Figure 10B).

**Figure 10:**
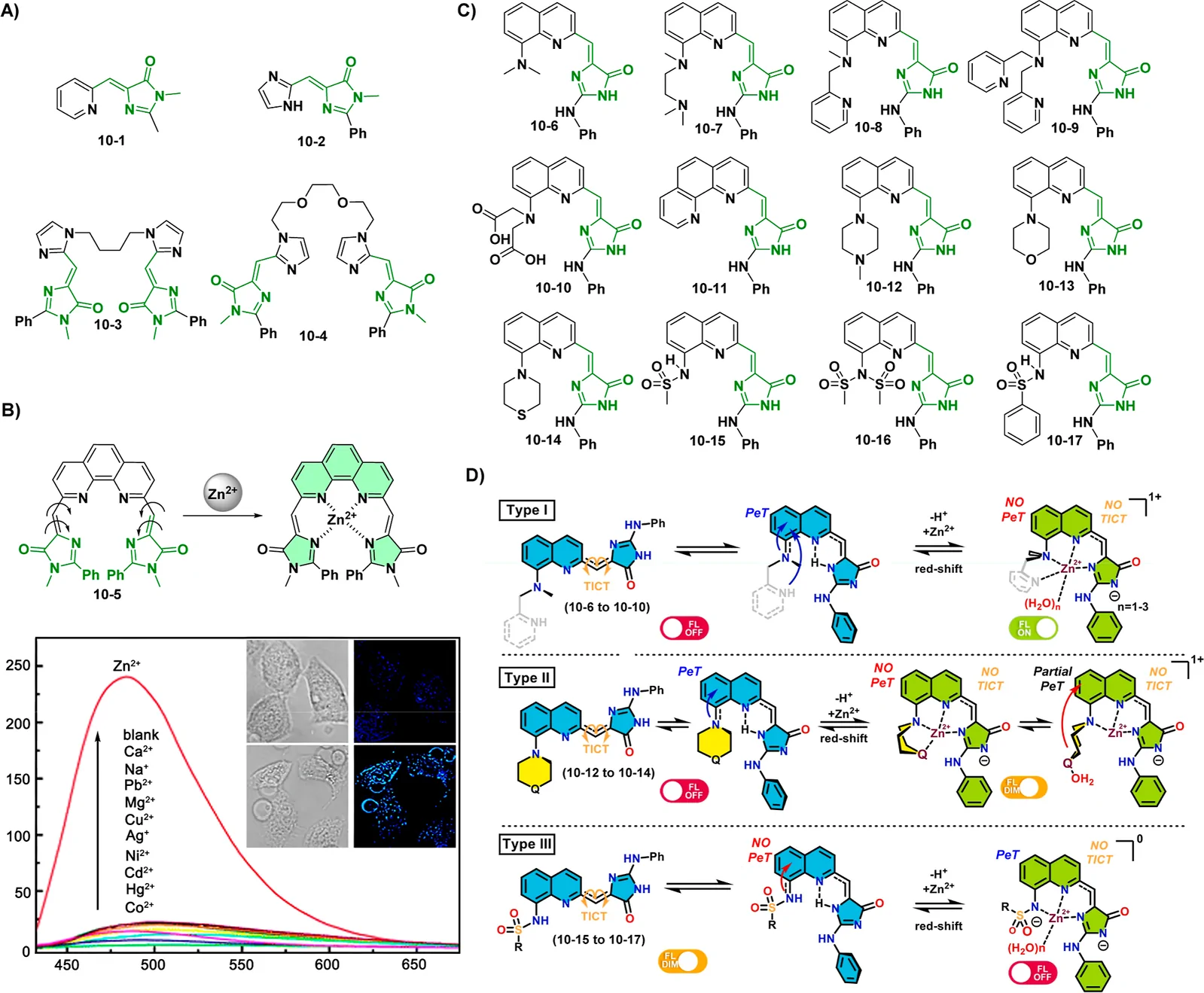
(A) Structures of **10-1** to **10-4**. (B) Sensing mechanism and emission spectra of **10-5** for detection of Zn^2+^. Inset: images of live HeLa cells incubated with **10-1** in the absence (top) and presence (bottom) of Zn^2+^, the original publication did not include scale bars in the reported images. (C) Structures of two-photon fluorescent chemosensors for Zn^2+^. (D) Three Zn^2+^ sensing mechanisms of probes. Panel B adapted with permission from ref 92 Copyright 2011 The Royal Society of Chemistry. Panel D adapted with permission from ref 93 Copyright 2023 the Authors, published by Elsevier B.V.

In 2024, Mucsi, Rózsa, Kovács, and co-workers introduced 12 GFP-chromophore-based chemosensors (**10-6** to **10-17**) that merge a Zn^2+^ chelator (8-aminoquinoline motif) with an imidazolinone core (Figure 10C).^93^ Notably, not all probes in this series exhibited a “turn on” response, with differential fluorescence activation/quenching mechanisms at play, including both twisted internal charge transfer (TICT) and photoinduced electron transfer (PeT). Additionally, these systems underwent three types of sensing mechanism, and they are therefore classed Type I–III (Figure 10D).^93^ Type I sensors (**10-6** to **10-10**) operate along the same principles as previously discussed: Zn^2+^ chelation restricts intramolecular rotation of the GFP-derived chromophore, leading to a bathochromic shift and a pronounced fluorescence “turn-on” response. These probes show high efficiency in Zn^2+^ detection, with good fluorescence enhancement and brightness. Two-photon absorption measurements further demonstrated that several of these Type I probes (notably **10-7**, **10-8**, and **10-9**) possess relatively high two-photon absorption cross sections (δ up to 340 GM, δ×Φ up to 5.36 GM), enabling effective fluorescence imaging of Zn^2+^ in live cells and brain slices. In contrast, Type II and III sensors exhibited less favorable properties. Type II probes (**10-12** to **10-14**), which contain saturated heterocyclic side chains, form relatively weak Zn^2+^ complexes. In these complexes, a coordinated water molecule can displace the distal heteroatom of the side chain, allowing the heterocycle to adopt its preferred chair conformation. The distal heteroatom is then proposed to continue mediating PeT quenching, accounting for the weak fluorescence turn-on and low brightness of the Type II probes. As a result, only weak fluorescence “turn-on” responses are observed. Conversely, Type III probes (**10-15** to **10-17**) display noticeable background fluorescence in their free form, but upon Zn^2+^ binding their emission decreases due to the promotion of PeT quenching, giving negative fluorescence enhancement factors and thus no useful sensing response.

### 4.4 Viscosity Detection and Imaging

As discussed throughout, the key factor modulating the fluorescence of GFP-chromophore analogues is free vibration and rotation causing nonradiative relaxation, thus weakening fluorescence. So far, several chemical mechanisms for rigidification to increase fluorescence have been presented, however the physical environment can also play a key role—in particular viscosity, whose effect is similar. Thus, GFP chromophore analogues can be used in designing fluorescent probes for sensing viscosity.

Intracellular viscosity is an important microenvironmental parameter for understanding biological systems and their disorders. Abnormal changes in viscosity have significant impact on the diffusion and transport of substances within cells, the interaction between biomolecules, signal transduction, and the diffusion of metabolites.^94,95^ As such, alterations in intracellular viscosity are strongly associated with a variety of diseases.^96,97^ Additionally, viscosity between different organelles varies widely, and so viscosity can also be a tool for organelle-targeted sensing. To better understand the effects of viscosity changes of cells, evaluation within specific organelles may be more meaningful than within the bulk cytoplasm.

Lysosomes serve as major signaling hubs within the cell, influencing fundamental processes such as membrane repair, energy metabolism, and inflammatory pathways.^98–100^ Therefore, the real-time monitoring of dynamic changes in lysosomal viscosity holds significant importance for the holistic management of diseases associated with lysosomes. In 2018, Huang et al. reported a lysosomal viscosity based probe **11-1** (Figure 11A), built around the usual extended **HBDI**-type scaffold with added morpholine side-chains.^37^ In low viscosity environments this probe exhibits weak fluorescence, with its emission fluorescence intensity at 515 nm increasing with viscosity (Figure 11A). This fluorescence response was found to be unaffected by pH, allowing reliable dynamic monitoring of lysosomal viscosity within cells through the fluorescence signal readout. However, the effectiveness of these viscosity probes is limited by factors such as sensitivity, response time, and the level of background luminescence noise, which can impact the accuracy of viscosity measurements.^101,102^ In contrast, Qian and colleagues developed **11-2** (Figure 11B), which is activated by acidic pH.^103^ Here, the electron-rich morpholine, reduces the brightness of the fluorophore by PeT quenching. On exposure to acidic pH, resulting in protonation this effect is reduced, restoring fluorescence, enabling pH-activatable lysosomal-targeted fluorescence with significantly reduced background fluorescence from outside of the lysosomes.

**Figure 11:**
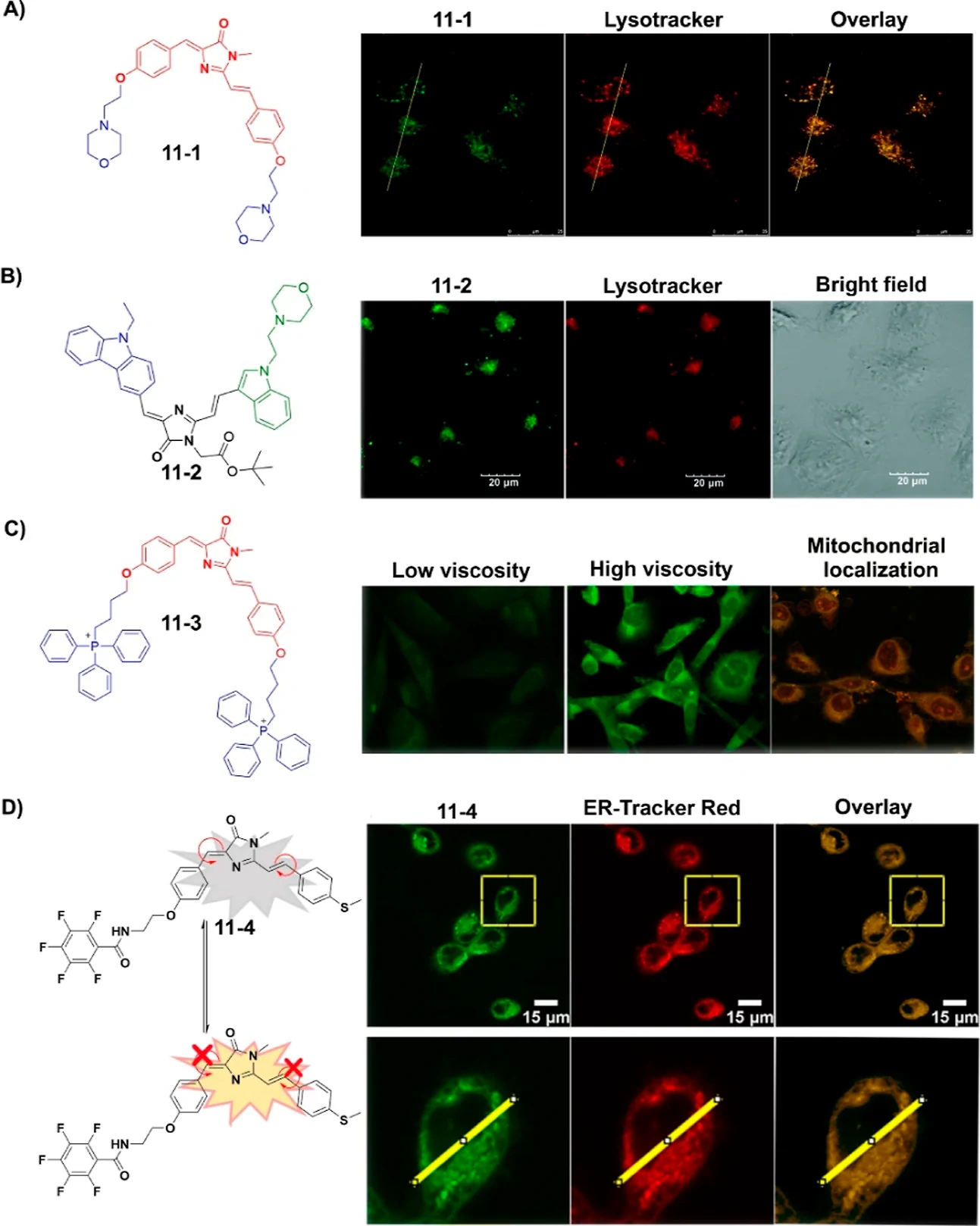
(A) Probe **11-1**. Fluorescence images of live MCF-7 cells costained with **11-1** and commercially available probe Lyso-Tracker Red. Scale bar 25 μm. (B) Probe **11-2**. Fluorescence images of Bel-7402 cells costained with **11-2** and Lyso-Tracker Red. Left to right: Confocal image of **11-2** in the green channel; Confocal image of Lyso-Tracker Red in the red channel; Bright-field image. Scale bar: 20 μm. (C) Probe **11-3**. Fluorescence images of HeLa cells costained with **11-3** and commercially available probe MitoTracker Deep Red FM. Left to right: Fluorescence images of cells with **11-3**; Fluorescence images of live cells stained with **11-3** after that the cells were pretreated with dexamethasone to increase the mitochondrial viscosity; Fluorescence merged images of cells costained with **11-3** and MitoTracker Deep Red FM after the cells were treated with dexamethasone for increasing the viscosity, the original publication did not include scale bars in the reported images. (D) Probe **11-4**. Fluorescence images of live MCF-7 cells costained with **11-4** and commercially available probe ER-Tracker Red. Top: whole images; bottom: enlarged regions of interest of the top lane. Scale bar: 15 μm. Panel A adapted with permission from ref 37 Copyright 2018 The Royal Society of Chemistry. Panel B adapted with permission from ref 103 Copyright 2020 The Royal Society of Chemistry and the Centre National de la Recherche Scientifique. Panel C adapted with permission from ref 104 Copyright 2021 American Chemical Society. Panel D adapted from ref 105 under a Creative Commons Attribution 3.0 Unported License (CC BY 3.0).

Another organelle of interest is the mitochondria, which is indispensable in regulating cell signaling pathways, metabolism, respiratory cycles, and apoptosis.^106,107^ Defects and dysfunction of the mitochondria are closely associated with the onset of many diseases such as cancer, diabetes, cardiovascular, and neurodegenerative diseases.^108,109^ Many classes of fluorescence based sensors have been previously reported for studying mitochondrial viscosity, incorporating BODIPY,^110,111^ hemicyanine,^112,113^ and rotors based on pyridine.^114^ Adding GFP chromophore analogues to this list, Cai et al. developed a mitochondria-targeted fluorescent probe **11-3** (Figure 11C), consisting of triphenylphosphonium groups attached to a **HBDI** core.^104^ This probe operates using the same “turn-on” principles as those discussed throughout, sensing viscosity in mitochondria through a restriction in rotation, while the triphenylphosphonium groups facilitate localization of the probe in mitochondria through their innate mitochondria-targeting ability.

Finally, viscosity has also been imaged using **HBDIs** in the endoplasmic reticulum (ER), a crucial multifunctional organelle in eukaryotic cells, responsible for lipid and steroid synthesis and metabolism, maintenance of Ca^2+^ homeostasis, and protein synthesis, folding, and modification.^115^ Studies have shown that ER stress coexists with autophagy and is closely related to cardiovascular diseases,^116^ Alzheimer’s disease,^117,118^ diabetes,^117^ and cancer,^119^ among many others. In 2022, Wu, James and Huang et al. developed a **HBDI**-based probe **11-4** for rapid monitoring of ER viscosity changes (Figure 11D).^105^ Here, a *para*-thioether styryl group extends conjugation to red shift the emission wavelength, and a pentafluorobenzene serves as the ER-targeting element. In a low-viscosity environments, free rotation leads to a low Φ of the probe, which increases sharply on admission into the ER due to high viscosity.

### 4.5 GFP Chromophore-based Probes in Other Applications

The (*Z*)-5-benzylideneimidazolinone scaffold exhibits exploitable excited-state reactivity that extends beyond fluorescence. In a representative example, Zhang X, Zhang L and Liu and co-workers developed a series of GFP-chromophore analogs that harness this excited-state reactivity for photosensitization and light-induced covalent cross-linking.^120^ Structural modulation by extending conjugation and introducing heavy atoms such as bromine and iodine enhanced triplet state reactivity and promoted reactive oxygen species generation. Among the derivatives, D6 (Figure 12A) exhibited the highest photo cross-linking efficiency toward aggregated proteins, with up to 77% monomer loss observed by SDS PAGE, while D2 exhibited the highest reactive oxygen species generation efficiency. KillerRed produced negligible reactive oxygen species under the same DCFH assay. Benefiting from their selective affinity for aggregated proteins, these probes, especially D6, enabled light dependent cross-linking of aggregated proteome from stressed cells, and LC MS/MS analysis revealed oxidation and cross-linking sites as well as interaction networks. These chromophores provide new tools to study protein aggregation mechanisms and hold promise for applications such as photodynamic therapy, chromophore assisted light inactivation, and photo induced polymerization.

**Figure 12:**
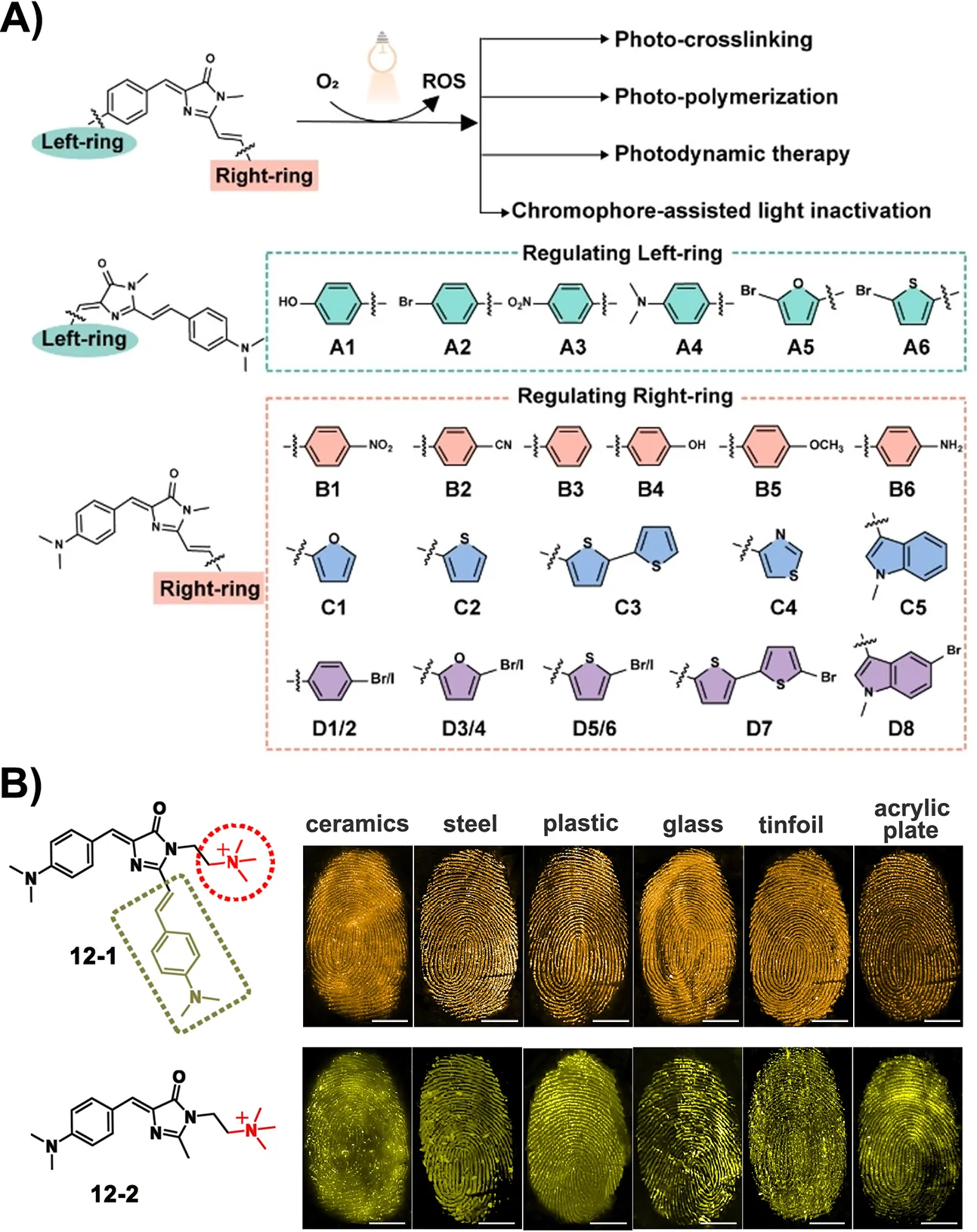
(A) Design strategy and structures of series of new GFP chromophore analogues with photosensitizing and photo cross-linking functions. (B) Chemical structures of synthesized **12-1** and **12-2** for latent fingerprints (LFPs). RGB color fluorescence photographs of LFPs on different substrates, with substrate colors removed using ImageJ, visualized by **12-1** and **12-2**, respectively (scale bars: 5 mm, under 445 nm irradiation). Panel A adapted with permission from ref 120 Copyright 2022 Wiley. Panel B adapted with permission from ref 121 Copyright 2024 The Authors, published by American Chemical Society.

**Figure 13:**
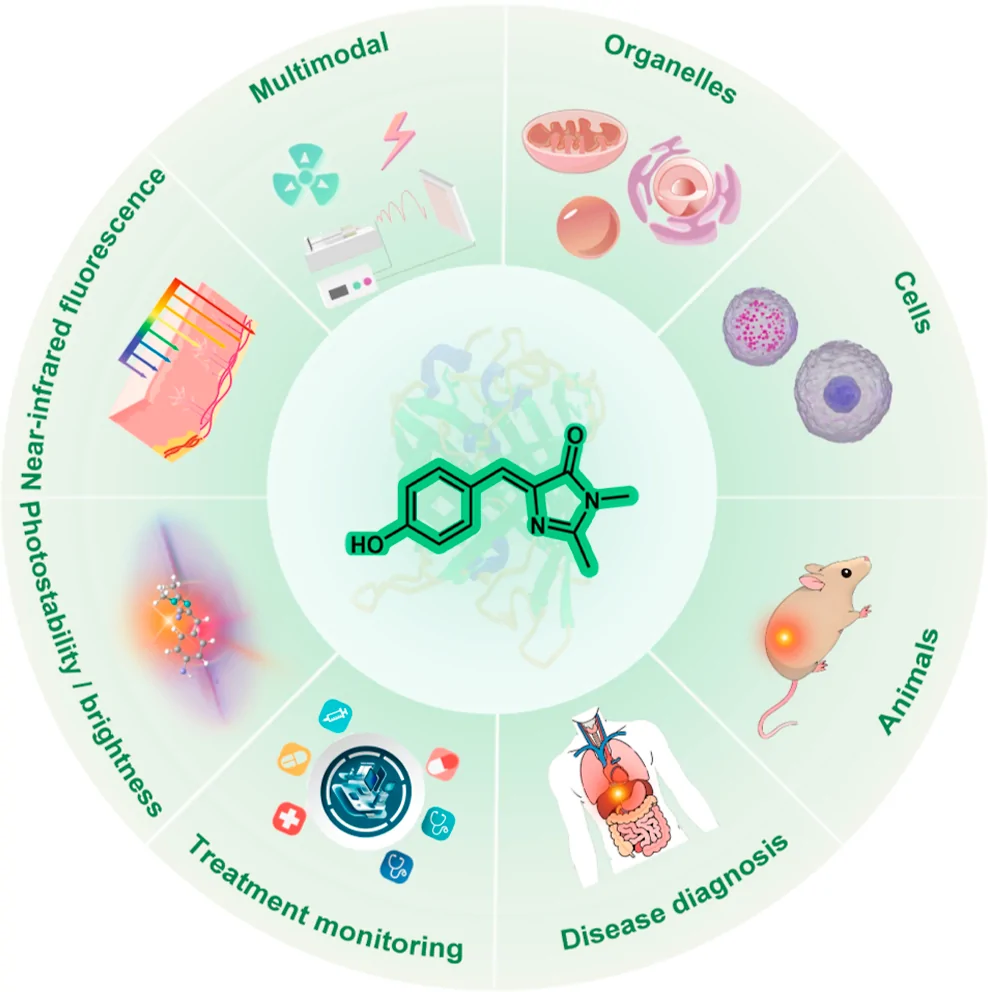
Prospective insights and potential research directions.

Recently in 2024, Wu, James, and Huang et al. extended the application of GFP chromophore analogues to the forensic sciences by developing two novel probes **12-1** and **12-2** (Figure 12B) for visualizing latent fingerprints (LFPs).^121^ These probes were constructed by attaching cationic groups (trimethylammonium) to a typical **HBDI**-derived system as well as a *p*-dimethylamine substituent at the benzylidene end to enhance fluorescence. Both probes performed well in visualizing LFPs on various objects such as ceramics, steel, plastic, glass, tinfoil, or acrylic plate. Starting from weak fluorescence due to intramolecular motion, the fluorescence intensity increased on exposure to LFPs thanks to selective noncovalent interactions with the biomolecules present in LFPs, enabling their visualization. Additionally, level 1–3 characteristics (from macroscopic pattern-type to microscopic details such as pores) of the LFPs could be clearly visualized. Alongside this level of detail, short tandem repeat analysis confirmed the probe does not cause damage to DNA, thus avoiding alteration of evidence, and enabling follow-up DNA analysis from LFP material after treatment with these probes. Furthermore, the low toxicity of these probes presents clear safety benefits for users.

## 5 Opportunities and Challenges

### 5.1 Advantages and Limitations of GFP Chromophore-Based Probes

As outlined throughout this perspective, the future of GFP chromophore analogues in sensing and imaging is promising (see Figure 13), primarily due to the synthetic versatility of the core scaffold. This chemical tractability enables relatively straightforward access to these systems and, more importantly, allows rational, precise, and relatively facile structural modification. Key photophysical parameters, including absorption and emission wavelengths, Stokes shift, and Φ can be systematically tuned. Functional groups can also be incorporated to enhance selectivity, such as recognition motifs for specific ions, proteins, or nucleic acids, to enable bioorthogonal labeling via HaloTag ligands or click chemistry handles, or to improve physical properties, including water solubility through sulfonate groups, membrane permeability via lipophilic substituents, and photostability through conformational locking, as required.

This synthetic approach is complementary to genetically encoded fluorescent proteins. At a molecular design level it offers a direct route to probe diversification and optimization that is not constrained by cellular expression, protein folding, and post-translational maturation. In addition, the compact size of GFP chromophore analogues reduces steric bulk relative to fluorescent proteins when conjugated to biomolecules, which may help to minimize interference with protein folding, trafficking, or function.^122^ However, reduced molecular size does not imply negligible perturbation. In practice, probe performance depends critically on the labeling site, linker design, and local microenvironment, and many reported analogues still suffer from limited membrane permeability. As a result, successful live-cell applications require careful probe engineering and delivery strategies. Existing applications of these systems are already wide-ranging, from dynamic tracing of different biological processes (e.g., protein–protein interaction) to visualization of intracellular viscosity changes.

Compared to common small molecule fluorophores such as coumarin, fluorescein, or rhodamine, GFP chromophore analogues are a new type of small molecular dyes whose fluorescence is modulated through restriction of their degrees of freedom. Microenvironments or target molecules that can mimic the β-barrel environment which constrains the rotation of the chromophore to elicit fluorescence can in principle be studied by these systems, since a “turn-on” response can be expected. This presents a new tool for the development of novel fluorescent probes, harnessing the biomimetic properties of these GFP chromophore analogues.

However, like most small organic fluorophores, this class of probes suffers from issues including photobleaching, low fluorescence brightness, and cell impermeability, etc. And although a broad range of structural variations are accessible, no single high-yielding general synthetic approach exists, requiring tailored individualized synthetic approaches for each new analogue, drastically slowing progress and providing a significant barrier to entry.

### 5.2 Prospective Insights and Potential Research Directions

#### 5.2.1 Conformation-Locked GFP Chromophore Analogues and Their Use in Constructing Probes for Biosensing and Bioimaging

Conformational locking has been demonstrated to significantly enhance the Φ and photostability of GFP chromophore analogues, with related probes showing great promise in biomacromolecule labeling. The efficacy of this strategy is rooted in the fundamental photophysics outlined in Sections 1 and 3: the fluorescence of the **HBDI** core is intrinsically quenched by rapid nonradiative decay through torsional motion around the exocyclic double bond and single bonds. Conformational locking, via strategies such as intramolecular hydrogen bonding, boron complexation, or steric constraint, directly suppresses these motions. This mimics the natural rigidification within the GFP β-barrel, forcing the chromophore into a planar, fluorescent state and closing the major nonradiative decay channels. In the future, torsional freedom of the fluorophore may be further restricted by introducing more complex steric hindrance or by exploiting metal coordination. For example, “semirigid” fluorophores based on transition metal complexes could combine partial conformational flexibility that allows adaptation to local microenvironments with suppression of nonradiative decay.

#### 5.2.2 The Development of Near-Infrared and Multiphoton GFP Chromophore-Based Probes

Fluorescence guided surgery with tumor targeted imaging agents have become important for identifying tumor margins during operations.^123^ Most fluorescent proteins emit in the visible region, which limits tissue penetration. The development of GFP chromophore inspired fluorophores that emit in the NIR region, particularly in the NIR II window, will markedly improve in vivo imaging resolution and penetration depth.^124^ Expanding the π-conjugated framework or introducing electron-donating or electron-withdrawing groups are feasible strategies to achieve red-shifted emission.^33,125^ At the same time, efficient two-photon (2P) or three-photon (3P) absorption probes combined with long-wavelength excitation can provide high-resolution imaging of deep tissues. Beyond existing 2P GFP based chemosensors,^93,126^ new analogues may enable 3P fluorescence imaging, as several GFP variants have already shown potential in this area.^127,128^ Thus, the construction of new GFP chromophore analogues for multiphoton fluorescence imaging represents a promising research direction.

#### 5.2.3 Design of Multimodal GFP Chromophore-Based Probes

Due to the limited depth of tissue penetration and imaging resolution, fluorescence imaging can be integrated with complementary imaging modalities to maximize their respective advantages. For example, fluorophores that can also chelate radionuclides would combine the high spatial resolution of fluorescence imaging with the quantitative properties of positron tomography.^97^ In addition, GFP chromophore analogues with acoustically or Raman active groups could enable the development of new photoacoustic or surface-enhanced Raman scattering probes, leveraging plasmonic nanostructures for signal amplification. Since it is difficult to use a mixture of various probes together in one dose to realize multimodal imaging due to the differences in sizes, pharmacokinetic properties etc., integrating multiple imaging modalities into one probe will endow these probes with both fluorescence based and additional modes of imaging. Thus, the development of single GFP chromophore-based probes by incorporating various functional groups could greatly expand the utility of GFP inspired chromophores in biomedical imaging.

#### 5.2.4 Advancing Multiorganelle Imaging with GFP-Chromophore Probes

A key challenge in single-probe multiorganelle imaging is how to distinguish different compartments using one molecular probe. Organelle differentiation can be achieved when the same probe generates organelle-dependent fluorescence signatures, such as distinct emission colors,^129^ fluorescence lifetimes,^130^ or environment-sensitive ratiometric responses.^131^ Such single-probe designs may also simplify spectral management by reducing the need for multiple independent dyes. In reported single-probe dual-organelle systems, such differentiation is commonly realized by combining dual-targeting capability with dual-channel fluorescence outputs, allowing one probe to be assigned to different organelles according to its compartment-dependent optical responses.^132^ Significantly, a disease is rarely the result of a single organelle failing. It is in fact the collapse of the communication network between organelles. Thus, the ability to simultaneously observe organelle interactions is crucial for understanding cellular functions and related diseases such as apoptosis, autophagy, or metabolic reprogramming.^133,134^

Therefore, probes that label multiple organelles simultaneously could help observe the “talking” (or interaction) between multiple organelles, providing better insight into organelle dysfunction, which is important for understanding specific disease mechanisms. Some fluorescent probes have been designed to provide a proof of concept for such approaches.^132^

Simultaneous organelle labeling enables the visualization of spatial proximity, temporal relationships, and dynamic organelle interactions.^135^

### 5.3 Conclusions and Prospects

Looking forward, GFP-inspired synthetic chromophores have evolved from structural mimics to functional optical tools, though clear gaps remain between their potential and routine biological application. Currently, these probes enable the environmentally sensitive detection of biomolecules, real-time reporting of protein misfolding/aggregation, organelle-specific viscosity mapping, and metal ion sensing with turn-on fluorescence. Their synthetic tunability allows emission color modulation and incorporation of targeting groups. However, several key challenges still limit their widespread adoption: (i) limited brightness and photostability compared to classic dyes like rhodamines; (ii) the lack of general, high-yielding synthetic routes for diverse analogues; (iii) insufficient selectivity in complex cellular environments; and (iv) the need for improved near-infrared and multiphoton versions for deep-tissue imaging. Addressing these issues through rational design, improved synthetic methodology, and systematic photophysical evaluations are essential in order to translate GFP-inspired chromophores into robust, widely useable probes for real-time, multicolor, and deep-tissue imaging in living systems.

## Supplementary Material



## Notes

It contains supporting data and respective Figures S1 and S2 (PDF)

## Abbreviations

GFP: green fluorescent protein
HBDI: *p*-hydroxybenzylideneimidazolidinone
HBRs: 4-hydroxybenzylidene-rhodanines
Φ: fluorescence quantum yield
SAM: *S*-adenosylmethionine
NIR: near-infrared
PeT: photoinduced electron transfer
FASTs: fluorescence-activating and absorption-shifting tags
ER: endoplasmic reticulum
LFPs: latent fingerprints
