# Supplementary material for: Lighting Up Biology: GFP-Inspired Fluorescent Probes for Sensing and Imaging
Source: J Am Chem Soc. 2026 Aug 11;148(33):35183–202. doi: 10.1021/jacs.5c16404 (PMC13523761; doi:10.1021/jacs.5c16404)
Supplement: Supplementary file 1 [file ja5c16404_si_001.pdf]

# Supporting Information

## Lighting Up Biology: GFP-Inspired Fluorescent Probes for Sensing and Imaging

Qianfang Qiu,[a‡] Luling Wu,[bc*‡] Robin R. Groleau,[d] Xuanyu Zhu,[a] Tony D. James,[ce*] and Chusen Huang[a*]

a. The Education Ministry Key Laboratory of Resource Chemistry, Shanghai Key Laboratory of Rare Earth Functional Materials, Shanghai Frontiers Science Center of Biomimetic Catalysis, and Shanghai Municipal Education Committee Key Laboratory of Molecular Imaging Probes and Sensors, Department of Chemistry, Shanghai Normal University, 100 Guilin Road, Shanghai 200234, China

b. State Key Laboratory of Analytical Chemistry for Life Science, School of Chemistry, Nanjing University, 163 Xianlin Avenue, Nanjing, 210023, China

c. Department of Chemistry, University of Bath, Bath BA2 7AY, U.K.

d. Department of Life Sciences, University of Bath, Bath BA2 7AY, U.K.

e. School of Chemistry and Chemical Engineering, Henan Normal University, Xinxiang 453007, China

*Email: wllcyl@126.com; t.d.james@bath.ac.uk; huangcs@shnu.edu.cn

## 1. Cellular imaging applications of pFAST fusions and splitFAST systems.

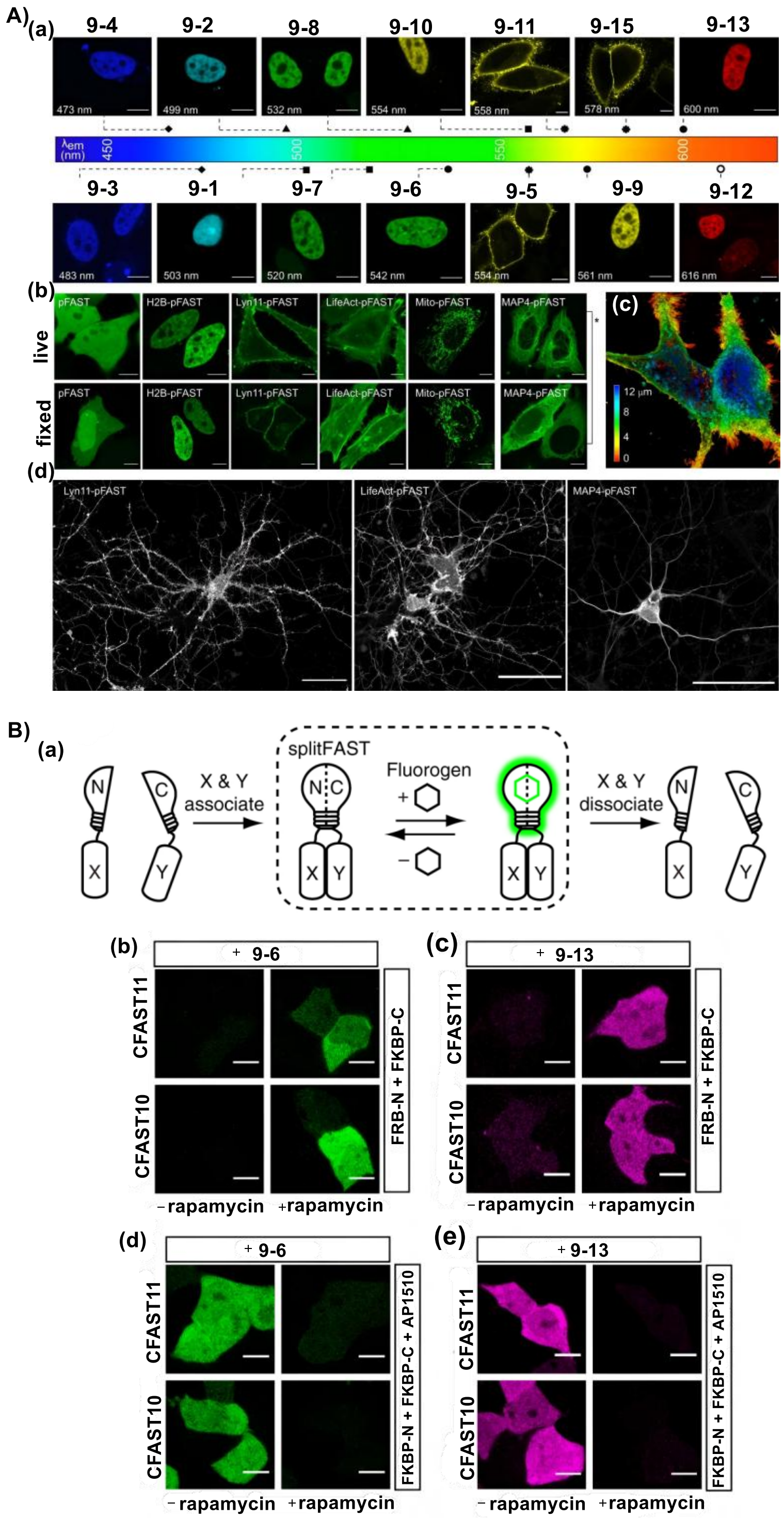


**Figure S1.** (A) Fluorescence images of pFAST fusions in various cellular localizations in mammalian cells and cultured neurons. (a) Fluorescence images of HeLa cells expressing pFAST fusions in the presence of various **HBR** derivatives listed in Figure 9B; (b) Fluorescence images of live and fixed HeLa cells expressing pFAST fused to: histone H2B, lyn11 (inner membrane-

targeting motif), LifeAct (actin-binding peptide domain), mito (mitochondrial targeting motif), and to microtubule-associated protein (MAP) 4 in the presence of 5 μM **9-7**; (c) Depth color coded three-dimensional reconstruction of live HeLa cells expressing lyn11-pFAST fusion in the presence of **9-10**; (d) Fluorescence images of live dissociated hippocampal neurons expressing pFAST fused to lyn11, LifeAct, and MAP4 in the presence of 10 μM **9-13**. Scale bars 50 μm. (B) splitFAST enables the observation of transient protein–protein interactions through the rapid and reversible binding to **HBR** analogs. (a) Design strategy; (b, c) HEK293T cells co-expressing FKBP-CFASTn (n = 10 or 11) and FRB-NFAST were labeled with 5 μM **9-6** (b) or 10 μM **9-13** (c) and imaged before and after the addition of 100 nM rapamycin; (d, e) AP1510-treated HEK293T cells co-expressing FKBP-NFAST and FKBP-CFASTn (n = 10 or 11) were labeled with 5 μM **9-6** (d) or 10 μM **9-13** (e) and imaged before and after the addition of 1 μM rapamycin. Scale bars 10 μm. Panels A and B adapted with permission from ref[1] and ref[2], Copyright Springer Nature.

**2. Spectral characterization of fluorophores 9-18 and 9-19.**

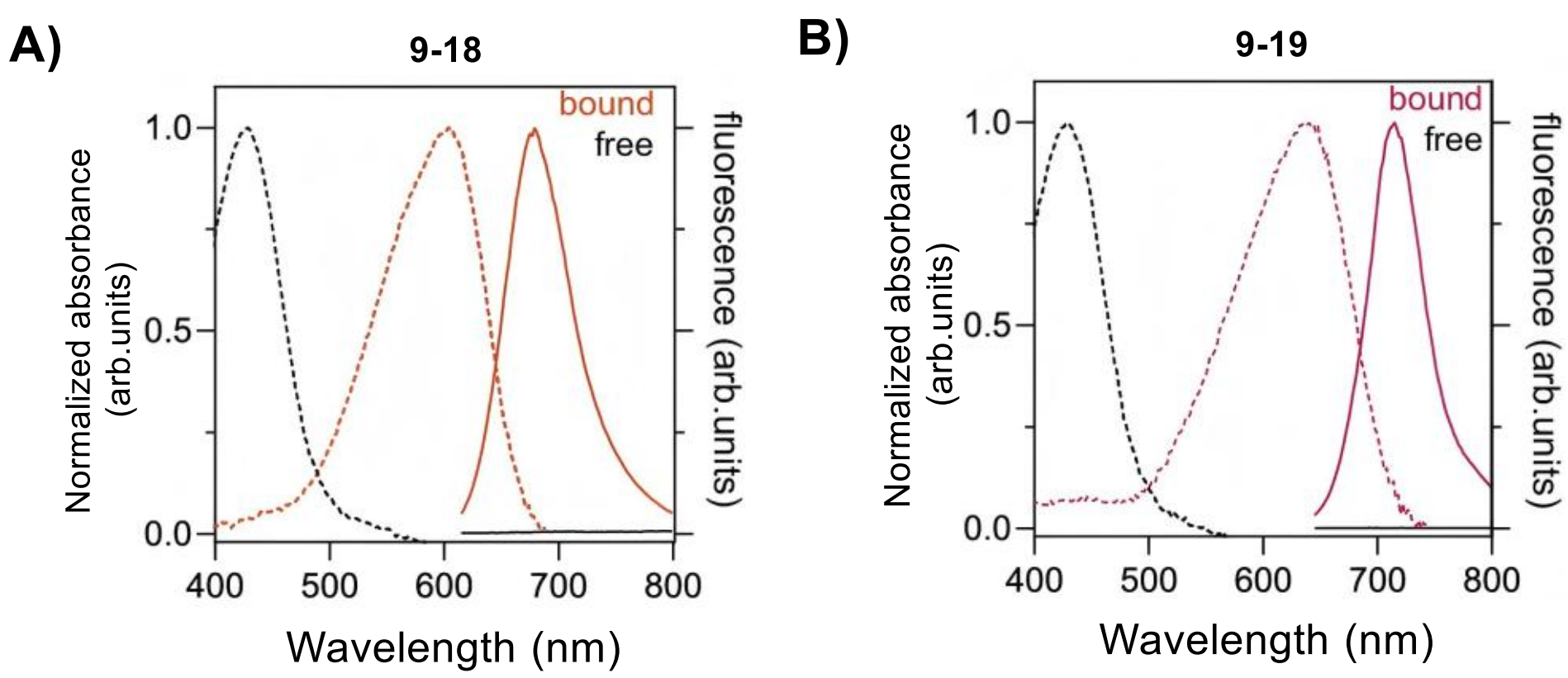


**Figure S2.** (A) Normalized absorption (dashed) and emission (solid) spectra of **9-18** in the free state (black) and bound to nirFAST (orange). (B) Normalized absorption (dashed) and emission (solid) spectra of **9-19** in the free state (black) and bound to nirFAST (dark red). Panels A and B adapted with permission from ref[3], Copyright Springer Nature.

## 2. References

1. Benaissa, H.; Ounoughi, K.; Aujard, I.; Fischer, E.; Goïame, R.; Nguyen, J.; Tebo, A. G.; Li, C.; Le Saux, T.; Bertolin, G.; Tramier, M.; Danglot, L.; Pietrancosta, N.; Morin, X.; Jullien, L.; Gautier, A., Engineering of a fluorescent chemogenetic reporter with tunable color for advanced live-cell imaging. *Nat. Commun.* **2021,** *12* (1), 6989.

2. Tebo, A. G.; Gautier, A., A split fluorescent reporter with rapid and reversible complementation. *Nat. Commun.* **2019,** *10* (1), 2822.

3. El Hajji, L.; Bunel, B.; Joliot, O.; Li, C.; Tebo, A. G.; Rampon, C.; Volovitch, M.; Fischer, E.; Pietrancosta, N.; Perez, F.; Morin, X.; Vriz, S.; Gautier, A., A tunable and versatile chemogenetic near-infrared fluorescent reporter. *Nat. Commun.* **2025,** *16* (1), 2594.
